# Myc targeted CDK18 promotes ATR and homologous recombination to mediate PARP inhibitor resistance in glioblastoma

**DOI:** 10.1038/s41467-019-10993-5

**Published:** 2019-07-02

**Authors:** Jian-Fang Ning, Monica Stanciu, Melissa R. Humphrey, Joshua Gorham, Hiroko Wakimoto, Reiko Nishihara, Jacqueline Lees, Lee Zou, Robert L. Martuza, Hiroaki Wakimoto, Samuel D. Rabkin

**Affiliations:** 1 Molecular Neurosurgery Laboratory and the Brain Tumor Research Center and Department of Neurosurgery, Massachusetts General Hospital and Harvard Medical School, Boston, 02114 MA USA; 2 Department of Neurosurgery, University of Minnesota Medical School, Minneapolis, 55455 MN USA; 3 The David H. Koch Institute for Integrative Cancer Research and Department of Biology, Massachusetts Institute of Technology, Cambridge, 02139 MA USA; 4 Department of Genetics, Harvard Medical School, Boston, 02115 MA USA; 5 Department of Pathology, Brigham’s and Women’s Hospital and Harvard Medical School, Boston, 02115 MA USA; 6 Department of Pathology, Massachusetts General Hospital and Harvard Medical School, Boston, 02114 MA USA; 7 Massachusetts General Hospital Cancer Center, Harvard Medical School, Charlestown, 02129 MA USA; 8 Brain Tumor Stem Cell Laboratory, Massachusetts General Hospital and Harvard Medical School, Boston, 02114 MA USA

**Keywords:** Double-strand DNA breaks, Homologous recombination, Targeted therapies, CNS cancer

## Abstract

PARP inhibitors (PARPis) have clinical efficacy in BRCA-deficient cancers, but not BRCA-intact tumors, including glioblastoma (GBM). We show that *MYC* or *MYCN* amplification in patient-derived glioblastoma stem-like cells (GSCs) generates sensitivity to PARPi via Myc-mediated transcriptional repression of *CDK18*, while most tumors without amplification are not sensitive. In response to PARPi, CDK18 facilitates ATR activation by interacting with ATR and regulating ATR-Rad9/ATR-ETAA1 interactions; thereby promoting homologous recombination (HR) and PARPi resistance. CDK18 knockdown or ATR inhibition in GSCs suppressed HR and conferred PARPi sensitivity, with ATR inhibitors synergizing with PARPis or sensitizing GSCs. ATR inhibitor VE822 combined with PARPi extended survival of mice bearing GSC-derived orthotopic tumors, irrespective of PARPi-sensitivity. These studies identify a role of CDK18 in ATR-regulated HR. We propose that combined blockade of ATR and PARP is an effective strategy for GBM, even for low-Myc GSCs that do not respond to PARPi alone, and potentially other PARPi-refractory tumors.

## Abstract (web summary)

PARP inhibitors are mainly used to treat BRCA1/2 mutated cancers. Here, the authors show that MYC amplified glioblastomas are sensitive to PARP inhibition due to CDK18 repression, which impairs ATR regulated homologous recombination repair, and that ATR inhibition sensitises glioblastomas to PARP inhibition.

## Introduction

Poly(ADP-ribose) polymerase (PARP) functions in a variety of DNA damage responses (DDRs)^1^. PARP1 and PARP2 contribute to the regulation of several DNA repair processes, including DNA single-strand break (SSB) repair and double-strand break (DSB) repair involving homologous recombination (HR), classical non-homologous end joining (NHEJ), and alternative NHEJ (A-NHEJ)^1^. PARP inhibition causes DSB accumulation during DNA replication, which is particularly cytotoxic in the absence of HR activity. This is the basis for the synthetic lethality of PARP inhibitors (PARPis) in cancers with HR deficiency, often due to mutations in *BRCA1/2* or other HR genes^2^. Clinical studies with PARPis demonstrated significant activity in breast and ovarian cancers with *BRCA1/2* germline mutations, and four PARPis (olaparib, rucaparib, niraparib and talazoparib) have been approved by the Food and Drug Administration (FDA)^3^. Despite this clinical promise, responses to PARPis are not universal, even in cancers carrying *BRCA1/2* mutations^2,3^. On the other hand, patients with cancers lacking characterized HR deficiencies sometimes benefit from PARPi combinations with DNA-damaging agents^3,4^. Currently, *BRCA1/2* status is the only patient stratification criteria. A better understanding of cellular signaling pathways and mechanisms governing response and nonresponse to PARPis is necessary to establish biomarkers predicting PARPi responses, overcome PARPi resistance, and treat PARPi refractory tumors.

Glioblastoma (GBM), the most malignant adult primary brain cancer and invariably lethal^5^, is a highly heterogeneous tumor, both between patients (inter-tumoral) and within a tumor (intra-tumoral)^6,7^. It is representative of tumors that lack driver mutations/deletions in *BRCA1/2* and are considered HR proficient. GBM contains GBM stem-like cells (GSCs), also referred to as brain tumor stem cells or initiating cells^8^, which are a sub-population of stem-like tumor cells that contribute to disease progression and recurrence, and thus are important therapeutic targets^9–11^. In the absence of validated markers, a consensus standardization of GSCs is lacking^11,12^. We define our GSCs as sphere-forming cells from tumor specimens that self-renew, differentiate, are highly tumorigenic, and recapitulate the patient’s tumor phenotype^10,13,14^. PARP1 is expressed in GBM^15^ and PARPis enhance temozolomide (TMZ), radiation, and oncolytic virus cytotoxicity in GSCs^16–18^. However, molecular signatures that correlate with GBM responsiveness to PARPi have not been defined.

Using a cohort of patient-derived GSCs, we screened for PARPi sensitivity and observed its association with overexpression/amplification of Myc transcription factors, MYC and MYCN (together hereafter Myc). We further discovered that Myc mediated PARPi sensitivity via direct transcriptional repression of cyclin-dependent kinase 18 (CDK18, PCTK3) alone. In GSCs, CDK18 promotes ATR activation and HR, rendering cells refractory to PARPi, making it a useful therapeutic target. Importantly, non-Myc, as well as Myc-amplified GSCs can be sensitized to PARPi by ATR inhibitor (ATRi). This established that targeting PARP together with the CDK18-ATR signaling axis induces lethality in a broad spectrum of GSCs, even in GSCs that do not respond to PARPi alone. Thus, despite GBM not exhibiting BRCAness^19^, our results suggest that PARPis alone can be used for the treatment of Myc-driven GBM and that the inhibition of both PARP and ATR is effective even in non-Myc-amplified GBM.

## Results

### Myc overexpression renders GSCs sensitive to PARPi

**Fig. 1:**
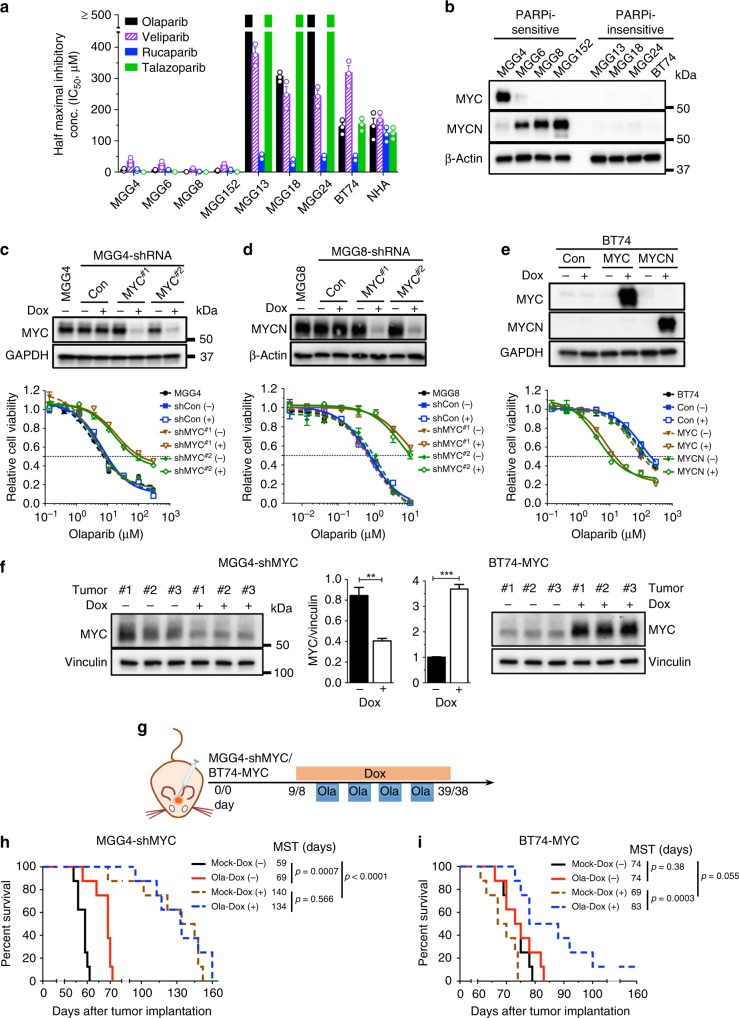
MYC/MYCN overexpression induces poly(ADP-ribose) polymerase inhibitor (PARPi) sensitivity in glioblastoma stem-like cells (GSCs). **a** Half maximal inhibitory concentration (IC_50_) of PARPis. GSCs were treated with the indicated PARPis for 6 days and cell viability was measured. Error bars depict mean ± SEM from three independent experiments in triplicate. **b** Representative western blot (*n* = 3) for the expression of MYC and MYCN in PARPi-sensitive and PARPi-resistant GSCs. β-Actin was the loading control. Proteins are listed on the left, molecular weight markers are indicated to the right. **c**–**e** Effect of MYC/MYCN knockdown or overexpression on PARPi sensitivity. (upper) Representative western blots (*n* = 3) showing doxycycline (Dox)-inducible MYC and MYCN knockdown (**c**, **d**; two independent short hairpin RNA sequences) or expression (**e**). Cells induced with (+) or without (−) Dox (1 μg/ml) for 4 days. Con scrambled sequence or empty vector control. GAPDH or β-Actin was the loading control. (lower) Olaparib dose–response curves (cell viability) for GSCs. Cells treated with olaparib in the presence (+) or absence (−) of Dox for 6 days, followed by MTS assay. Data normalized to relevant control and mean ± SEM, three independent experiments performed in triplicate. **f** Western blots showing MYC knockdown in MGG4-shMYC#1 (left) and overexpression in BT74-MYC (right) intracerebral xenografts treated with (+) or without (−) Dox (1 mg/ml) for 10 days. Vinculin was the loading control. (Center) Quantification of the MYC levels from western blots. Mean ± SEM. **p < 0.01, ***p < 0.001, *t* test. **g** Treatment schedule for **h**, **i**. Dox (1 mg/ml) was given from 3 days before to 3 days after olaparib (Ola, 50 mg/kg, 4 cycles), with days listed for MGG4-shMYC and BT74-MYC, respectively. **h**, **i** Kaplan–Meier survival curves of mice bearing orthotopic MGG4-shMYC#1 (**h**) or BT74-MYC (**i**) xenografts treated with Ola or vehicle (Mock) in the presence (+) or absence (−) of Dox as in **g**. MST median survival time. Vertical lines indicate *p* value comparisons (log-rank test)

PARPi cytotoxicity was examined in a cohort of patient-derived GSCs^10^. Our previous study^18^ and current data (Fig. 1a) showed that GSCs generally fall into two classes regarding PARPi sensitivity: highly sensitive to olaparib with half maximal inhibitory concentration (IC_50_) < 10 μM (MGG4, MGG6, MGG8, and MGG152) or insensitive, with IC_50_ > 100 μM (MGG13, MGG18, MGG24, and BT74), greater than maximal plasma concentration^20^, while normal astrocytes (NHA) were insensitive (Fig. 1a). All cells expressed active PARP (Supplementary Fig. 1a). Similar differences in sensitivity were observed with three other PARPis approved or in clinical trial: veliparib, rucaparib, and talazoparib (Fig. 1a). We selected the first FDA-approved PARPi, olaparib, as the mainstream compound for our subsequent studies.

Based on previous genetic analysis of some of these GSCs, we noted that all PARPi-sensitive GSCs tested here have *MYC* or *MYCN* amplification^10,21,22^, so we examined whether this might contribute to PARPi sensitivity. None of the PARPi-insensitive GSCs had detectable Myc expression (Fig. 1b). We also examined matched patient-derived serum-cultured GBM cells (ScGCs^23^ or DGCs^14^). In contrast to MGG4 and MGG8 GSCs, the matched ScGCs did not express MYC or MYCN (Supplementary Fig. 1a) and were much less sensitive to olaparib (Supplementary Fig. 1b). To test whether MYC or MYCN is responsible for PARPi sensitivity in GSCs, we used doxycycline (Dox)-inducible short hairpin RNA (shRNA) lentivirus to transduce GSCs and transiently knock down MYC in MGG4 (MGG4-shMYC) and MYCN in MGG8 (MGG8-shMYCN) (Fig. 1c, d). MYC/MYCN knockdown suppressed cell growth in both GSCs (Supplementary Fig. 1c, d), indicating a role for Myc in driving proliferation. MYC/MYCN knockdown greatly reduced the sensitivity of MGG4 and MGG8 to PARPi, with over nine-fold increases in IC_50_ (Fig. 1c, d (lower), Supplementary Table 1). Conversely, Dox-induced overexpression of MYC or MYCN in non-Myc GSCs (BT74 and MGG18) did not alter proliferation overall (Supplementary Fig. 1d–g) but rendered insensitive GSCs responsive to PARPi, decreasing IC_50_ by about ten-fold in BT74 (Fig. 1e (lower)) and MGG18 (Supplementary Fig. 1h, Supplementary Table 1). Cell cycle analysis showed that MYC knockdown in MGG4 reduced the proportion of S-phase cells and increased G1 cells (Supplementary Fig. 2a), while overexpression of MYC in BT74 and MGG18 did not alter cell cycle profiles (Supplementary Fig. 2b, c). Therefore, Myc-induced sensitivity to PARPi is not due to changes in the cell cycle.

We next evaluated the effect of Myc expression on PARPi responses in vivo. Systemic administration of Dox induced knockdown (MGG4-shMYC) or overexpression (BT74-MYC) of MYC after intracerebral tumors were established (Fig. 1f). In MGG4 without MYC knockdown, PARPi significantly prolonged mouse survival compared with vehicle (Fig. 1g, h), as we described previously^18^. Transient MYC knockdown for 31 days greatly slowed tumor growth and abrogated the survival benefit of olaparib (Fig. 1h), consistent with in vitro results. In PARPi-insensitive BT74, there was no difference in survival between PARPi- and vehicle-treated groups (Fig. 1g, i), as we described previously^18^; however, MYC overexpression somewhat accelerated BT74 tumor growth (not significantly) and tumors now became responsive to olaparib (Fig. 1i). Thus expression of Myc in GSCs induces sensitivity to PARPi in vitro and in vivo.

### RNA sequencing reveals Myc repression of CDK18 expression

**Fig. 2:**
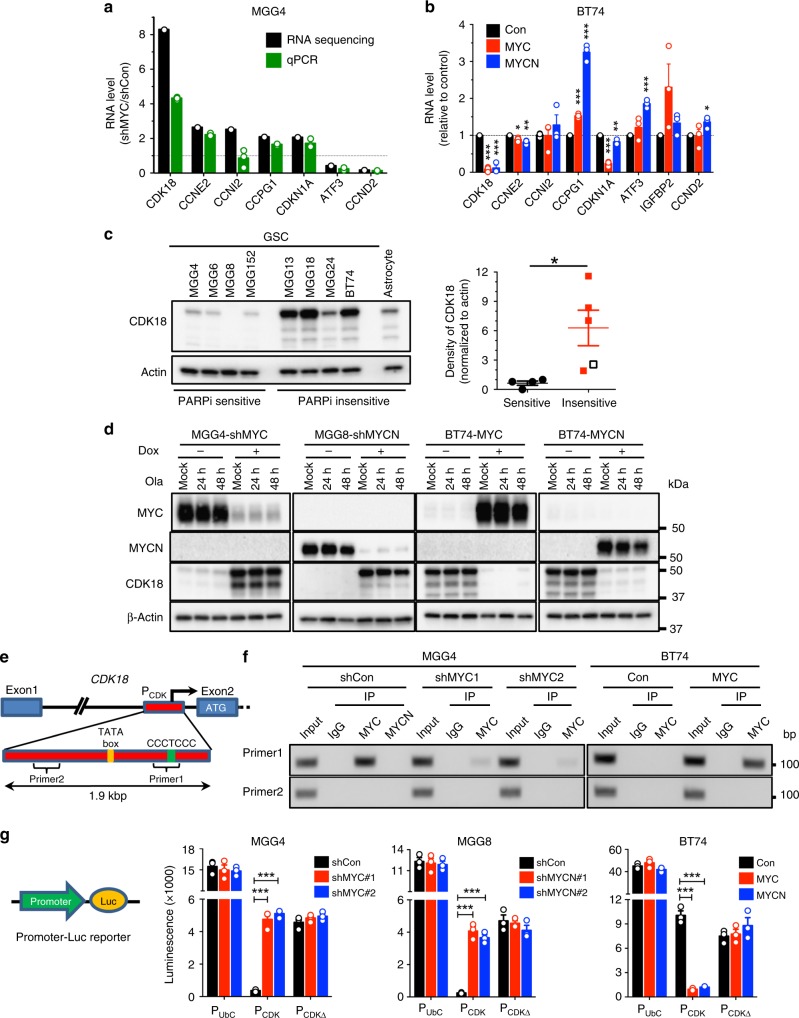
RNA sequencing reveals Myc transcriptional repression of cyclin-dependent kinase 18 (CDK18). **a** Fold change in RNA of the indicated genes in MGG4-shRNA-MYC (shMYC#1) over MGG4-shRNA-control (shCon) after olaparib treatment under doxycycline (Dox), as in Supplementary Fig. 3a. RNA was sequenced or subjected to quantitative reverse transcriptase–polymerase chain reaction (qRT-PCR; *n* = 3). **b** Fold change in RNA (qRT-PCR; n = 3) of the indicated genes in BT74-MYC and BT74-MYCN over control after Dox treatment for 6 days. Mean ± SEM. *p < 0.05, **p < 0.01, ***p < 0.001, *t* test. **c** (left) Representative western blot showing CDK18 expression in Myc-amplified glioblastoma stem-like cells (GSCs) (MGG4, MGG6, MGG8, and MGG152), non-Myc GSCs (MGG13, MGG18, MGG24, and BT74), and normal human astrocytes. Same membranes and β-Actin the loading control as in Supplementary Fig. 1a. (right) Quantification of CDK18 from western blot on left (empty square, astrocytes). *p < 0.05, *t* test. **d** Representative western blots (*n* = 3) showing MYC, MYCN, and CDK18 expression in MYC or MYCN knockdown (sh, shRNA sequences #1) or overexpressing GSCs treated with mock or Ola (10 μM) for 24 or 48 h, with (+) or without (−) Dox. β-Actin was the loading control. Proteins are listed on the left, molecular weight markers are indicated to the right. **e** Diagram of *CDK18* exon1–exon2 with intragenic promoter (P_CDK_), ATG, predicted TATA box, Myc-repressive motif CCCTCCC, and PCR primer targeting (primer1) or non-targeting (primer2) this motif. **f** Products of chromatin immunoprecipitation–PCR showing MYC, but not MYCN, specifically binding to its motif in MGG4 with and without shMYC (two independent sequences) and BT74 with and without MYC overexpression. DNA marker size is indicated on the right. **g** (left) Diagram of promoter (indicated in **f**) luciferase (Luc) reporter. (right) Expression of luciferase driven by human ubiquitin C promoter (P_UbC_) as control, CDK18 promoter (P_CDK_), or P_CDK_ lacking Myc-repressive motif (P_CDKΔ_) in GSCs with MYC/MYCN knockdown (sh; two independent sequences) or overexpression. shCon scrambled sequence, Con empty vector. ***p < 0.001, *t* test. Mean ± SEM

A primary function of Myc family transcription factors is to globally regulate gene expression, either activate or repress^24^. To gain insights into how Myc regulates PARPi sensitivity in GSCs, we performed RNA sequencing of MGG4 cells in the presence and absence of MYC and/or PARPi treatment (Supplementary Fig. 3a). Pathway enrichment analysis showed that olaparib distinctly downregulated several non-overlapping gene sets (e.g., translation, splicing and RNA processing, and WNT signaling) in MGG4-shControl cells (MYC intact) (Supplementary Fig. 3b). In contrast, in MYC knockdown cells, olaparib upregulated a greater number of gene sets involved in DDR and replication pathways (Supplementary Fig. 3b), suggesting that MYC suppression of these pathways may contribute to PARPi sensitivity. We then surveyed DDR genes and identified seven potentially interesting targets where MYC knockdown changed transcript levels over two-fold with statistical significance (*p* < 0.05, Chi-square test) and normalized values for shCon or shMYC over 1 (5: upregulated and 2: downregulated) (Fig. 2a, Supplementary Fig. 3c).

Quantitative reverse transcriptase–polymerase chain reaction (qRT-PCR) analysis validated MYC knockdown-induced regulation of six of these targets in MGG4 (Fig. 2a). In BT74, MYC and MYCN overexpression downregulated CDK18, CCNE2, and CDKN1A (Fig. 2b), consistent with Myc repression of these genes in GSCs. Among these, MYC/MYCN-mediated repression was greatest for CDK18. All non-Myc GSCs and normal astrocytes expressed CDK18 (Fig. 2c), which was barely detectable in Myc-amplified GSCs (Fig. 2c). To demonstrate that CDK18 expression was negatively regulated by Myc, we knocked down MYC/MYCN (MGG4-shMYC and MGG8-shMYCN), which greatly increased CDK18 protein, and overexpressed MYC/MYCN in BT74, which markedly reduced CDK18 protein (Fig. 2d). Olaparib treatment did not alter Myc or CDK18 levels (Fig. 2d). The effects of MYC and MYCN were indistinguishable, with Myc transcription factors selectively and potently repressing CDK18 in GSCs at both the RNA and protein levels. A minority of patients in the The Cancer Genome Atlas GBM dataset had MYC, MYCN, or CDK18 amplifications or altered mRNA levels, and there was no overlap between amplification of CDK18 and MYC or MYCN, suggesting a negative association of CDK18 and MYC or MYCN (Supplementary Fig. 4).

### Myc binds to CDK18 promoter and represses transcription

Next we asked whether Myc-mediated repression of CDK18 mRNA is due to direct Myc transcriptional regulation. Exon1 of the *CDK18* gene is entirely within the 5′-untranslated region and multiple promoter sites are postulated to be present upstream of exon1 and exon2 [https://genome.ucsc.edu/cgi-bin/hgGateway]. Using MYC and MYCN chromatin immunoprecipitation (ChIP)–PCR and GSCs with or without shMYC or MYC overexpression, we show that MYC and MYCN specifically bind to DNA regulatory sequences upstream of exon2 that contain a Myc-repressive binding motif, CCCTCCC (Fig. 2e, f, Supplementary Fig. 5)^25^. To determine whether this DNA region is a promoter that Myc represses, luciferase reporter vectors were constructed and transduced into GSCs. This DNA region has promoter activity that MYC and MYCN potently inhibit (Fig. 2g). A mutant promoter sequence lacking the CCCTCCC element (P_CDKΔ_) was not subject to Myc repression, indicating that Myc specifically binds to this site to repress transcription (Fig. 2g). Thus Myc regulates CDK18 transcript and protein levels via binding to a Myc repressive motif in *CDK18* that represses transcription.

### CDK18 loss mediates Myc-induced PARPi sensitivity in GSCs

**Fig. 3:**
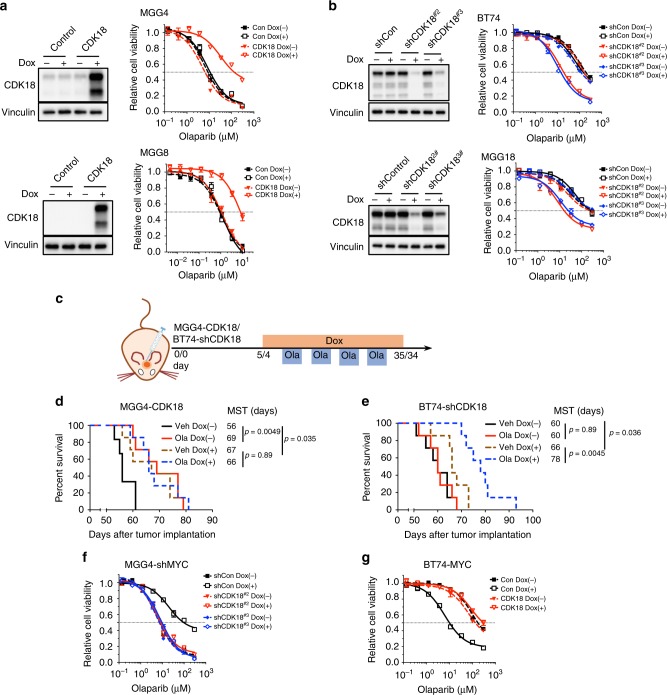
Cyclin-dependent kinase 18 (CDK18) regulates MYC/MYCN-induced poly(ADP-ribose) polymerase inhibitor sensitivity in glioblastoma stem-like cells. **a**, **b** Effect of CDK18 expression on olaparib sensitivity. (left) Western blots showing CDK18 overexpression or knockdown with (+) or without (−) doxycycline (Dox). (right) Olaparib dose–response curves. **a** MGG4-control (Con)/MGG4-CDK18 (upper) and MGG8-control (Con)/MGG8-CDK18 (lower). **b** BT74-shControl (shCon)/BT74-shCDK18 (upper) and MGG18-shControl (shCon)/MGG18-shCDK18 (lower) (shCDK18; two independent sequences), with (+) or without (−) Dox. **c** Treatment schedule for **d**, **e**. Details as in Fig. 1g. **d**, **e** Kaplan–Meier survival curves of mice bearing orthotopic MGG4-CDK18 (**d**) or BT74-shCDK18#2 (**e**) xenografts treated with olaparib (Ola) or vehicle (Veh) with (+) or without (−) Dox. MST median survival time. Vertical lines indicate *p* value comparisons (log-rank test). **f**, **g** Olaparib dose responses in MGG4 with knockdown of MYC alone (MGG4-shMYC-shCont) and with CDK18 (MGG4-shMYC-shCDK18 (two independent sequences) (**f**) or BT74-overexpressing MYC alone (BT74-MYC-Con) and with CDK18 (BT74-MYC-CDK18) (**g**), with (+) or without (−) Dox. Western blots are shown in Supplementary Fig. 6. Data normalized to control and mean ± SEM, representative of three independent experiments performed in triplicate

We next determined whether CDK18 contributes to Myc-induced PARPi sensitivity in GSCs. CDK18 overexpression converted PARPi-sensitive MGG4 and MGG8 to PARPi-insensitive cells with 16- and 7-fold increases in IC_50_ (Fig. 3a, Supplementary Table 2), similar to that seen after MYC/MYCN knockdown (Fig. 1c, d), which caused upregulation of endogenous CDK18 (Fig. 2d). Conversely, CDK18 knockdown in PARPi-insensitive BT74 and MGG18 decreased the IC_50_ by over nine-fold (Fig. 3b, Supplementary Table 2). To verify the effect of CDK18 in vivo, we induced its overexpression (MGG4-CDK18) or knockdown (BT74-shCDK18) in intracerebral tumors (Fig. 3c**)**. In MGG4 tumors, CDK18 overexpression slowed tumor growth and abrogated PARPi responsiveness (Fig. 3d). CDK18 knockdown in BT74 slowed tumor growth but rendered tumors responsive to PARPi (Fig. 3e).

To confirm that CDK18 is the mediator of Myc-induced PARPi sensitivity and is acting downstream of Myc, we knocked down CDK18 in MGG4-shMYC GSCs (Supplementary Fig. 6a), which abrogated PARPi insensitivity arising from MYC knockdown (Fig. 3f). Conversely, when CDK18 was overexpressed in BT74-MYC GSCs (Supplementary Fig. 6b), PARPi resistance was recovered (Fig. 3g). CDK18 manipulation similarly countered MYCN effects in MGG8-shMYCN and BT74-MYCN GSCs (Supplementary Fig. 6c, d). These data demonstrate that Myc sensitizes GSCs to PARPi by suppressing CDK18 expression and that CDK18 overrides the effects of Myc and is downstream.

### Myc inhibits HR via repressing CDK18

**Fig. 4:**
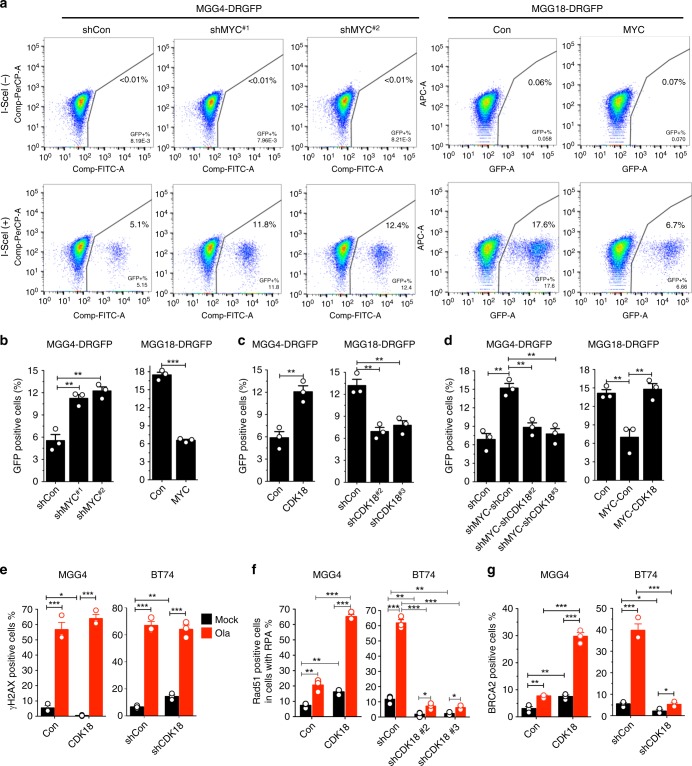
MYC inhibits and cyclin-dependent kinase 18 (CDK18) promotes homologous recombination (HR) in glioblastoma stem-like cells (GSCs). **a** Representative flow cytometry plots showing HR in MGG4-DRGFP expressing shRNA-Control (shCon) or shRNA-MYC (shMYC; two independent sequences) and MGG18-DRGFP expressing empty vector (Con) or MYC. GSCs were induced with doxycycline for 6 days before infection with lentivirus with (+) or without (−) I-SceI expression, followed by flow cytometry to measure green fluorescent protein (GFP)-positive cells (right quadrant) at 5 days after infection. **b** HR efficiency (percentage of GFP^+^) from **a** and two additional independent experiments. **c** HR efficiency in MGG4-DRGFP-CDK18 or MGG18-DRGFP-shCDK18 (two independent sequences). Western blot and representative fluorescence-activated cell sorting (FACS) plots shown in Supplementary Fig. 7. **d** HR efficiency in MGG4- or MGG18-DRGFP with double knockdown or overexpression of MYC and CDK18. Western blot and representative FACS plots shown in Supplementary Fig. 8. **e**–**g** Quantification of immunofluorescence staining of γH2AX (**e**), Rad51 positive foci in RPA positive (**f**), and BRCA2 (**g**) foci in GSCs with CDK18 overexpression or knockdown (sh) (shCDK18 #2 in **e**, **g**). Cells were treated with olaparib (Ola; 15 μM) or mock for 12 h before staining. Cell with ≥5 foci/cell were counted as positive. Representative images shown in Supplementary Fig. 9. Mean ± SEM, from three independent experiments. *p < 0.05, **p < 0.01 and ***p < 0.001, *t* test

One of the mechanisms underlying PARPi sensitivity is synthetic lethality due to HR deficiency (BRCAness)^19^. To evaluate HR, we stably transfected GSCs with HR reporter plasmid DRGFP and induced DSBs by lentivirus-mediated transduction of endonuclease I-SceI. PARPi-insensitive MGG18 (Con) has much higher levels of basal HR than MGG4 (shCon) (Fig. 4a, b). In MGG4, MYC knockdown (Supplementary Fig. 7a) increased HR efficiency about two-fold (Fig. 4a, b), despite the reduction in S-phase cells (Supplementary Fig. 2a), while MYC overexpression in MGG18 (Supplementary Fig. 7a) reduced HR by 62% (Fig. 4a, b), in the absence of changes in S-phase cells (Supplementary Fig. 2b, c). Conversely, CDK18 overexpression increased, and knockdown decreased HR efficiency (Fig. 4c, Supplementary Fig. 7b, c). As with PARPi sensitivity, CDK18 knockdown counteracted MYC-knockdown-mediated increases in HR in MGG4 (Fig. 4d, Supplementary Fig. 8a, b) and CDK18 overexpression reversed MYC-induced HR inhibition in MGG18 (Fig. 4d, Supplementary Fig. 8c, d).

To further evaluate the effects of CDK18 on PARPi-induced HR repair of DSBs, we used immunofluorescence to quantify γH2AX foci, a marker of DSBs and required for the assembly of DNA repair proteins^26^, and two key HR effectors: Rad51 foci in S/G2-phase cells, which are marked by RPA foci^27^ and BRCA2 foci, which recruit Rad51 to ssDNA or DSBs. At 12 h post olaparib treatment, both PARPi-sensitive MGG4 and PARPi-insensitive BT74 GSCs showed a large increase in γH2AX (Fig. 4e, Supplementary Fig. 9a). This was not affected by alterations in CDK18 levels (Fig. 4e, Supplementary Fig. 9a). In contrast, BT74 exhibited a much greater increase in BRCA2-positive cells and the proportion of RPA foci-positive cells that were Rad51 foci positive, indicative of HR repair, than MGG4 after olaparib (Fig. 4f, g, Supplementary Fig. 9b, c). CDK18 overexpression increased and CDK18 silencing decreased BRCA2 and Rad51 foci–RPA foci-positive cells (Fig. 4f, g, Supplementary Fig. 9b, c). Because RPA foci only form during S and G2 phases^27^, this indicates that CDK18 promotes Rad51 focus formation during S and G2 phases. Even in the absence of PARPi, the proportion of Rad51 and BRCA2 foci-positive cells were increased or decreased by CDK18 expression or knockdown, respectively (Fig. 4f, g). This suggests that CDK18 promotes BRCA2 localization at DSBs and thus Rad51-mediated HR. Collectively, MYC restrains HR through repression of CDK18, and CDK18 promotes HR even in the presence of MYC.

### Myc and CDK18 oppositely affect DDRs

**Fig. 5:**
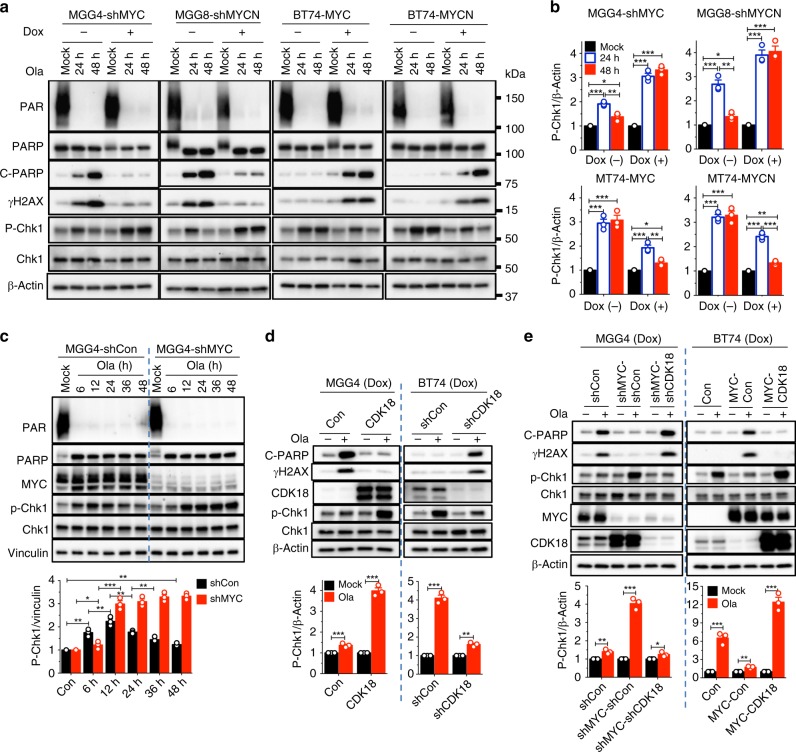
Myc inhibits and cyclin-dependent kinase 18 (CDK18) promotes ATR signaling in poly(ADP-ribose) polymerase inhibitor-treated glioblastoma stem-like cells (GSCs). **a** Representative western blots (*n* = 3) showing olaparib-induced DNA damage response (DDR) in GSCs with knockdown (sh) or overexpression of MYC and MYCN. Cells treated with mock or olaparib (Ola, 10 μM) for 24 and 48 h and with (+) or without (−) doxycycline (Dox). Same membranes and β-Actin control as in Fig. 2d. **b** Quantification of p-Chk1 levels determined from **a** and two additional independent experiments. Normalized to β-Actin. **c** (upper) Representative western blot (*n* = 3) showing kinetics (hours indicated) of p-Chk1 in olaparib-treated (10 μM) MGG4-shMYC or MGG4-shCon, with Dox. (lower) Quantification of p-Chk1 levels normalized to Vinculin from (upper) and two additional independent experiments. **d** (upper) Representative western blots (*n* = 3) showing olaparib-induced DDR in MGG4 with CDK18 overexpression or BT74 with CDK18 knockdown after treatment for 48 h with Dox. (lower) Quantification of p-Chk1 levels from (upper) and two additional independent experiments. With (+) or without (−) olaparib. **e** (Upper) Representative western blots (*n* = 3) showing olaparib-induced DDR in MGG4 with knockdown of MYC alone (MGG4-shMYC-shCon) and with CDK18 (MGG4-shMYC-shCDK18) or in BT74 overexpressing MYC alone (BT74-MYC-Con) and with CDK18 (BT74-MYC-CDK18), treated as in **d**. (lower) Quantification of p-Chk1 from (upper) and two additional independent experiments, normalized to β-Actin. *p < 0.05, **p < 0.01 and ***p < 0.001, *t* test. Mean ± SEM

Since Myc and CDK18 inversely modulate HR and PARPi sensitivity, we examined their effects on DDR. Silencing of Myc in MGG4 and MGG8 GSCs (Fig. 2d) markedly increased DNA repair, indicated by reduced DSBs (γH2AX) and apoptosis (c-PARP) after olaparib treatment (Fig. 5a). Conversely, MYC or MYCN overexpression in BT74 GSCs (Fig. 2a) robustly increased γH2AX and c-PARP upon olaparib treatment (Fig. 5a). We observed that these changes in DNA repair and cell death were linked with the kinetics of Chk1 activation, a key DDR effector and ATR substrate (Fig. 5a–c; p-Chk1). In MYC-overexpressing GSCs (MGG4-shCon or MGG4-shMyc/Dox− and BT74-Myc/Dox+), olaparib-induced p-Chk1 peaked around 12 h post-treatment and then decreased to almost basal levels at 48 h (Fig. 5a–c). This was not due to changes in total Chk1 levels (Fig. 5a, c). In contrast, in non-Myc BT74 or MGG4-shMYC GSCs that resist olaparib-induced DNA damage and toxicity, strong activation of Chk1 was sustained for at least 48 h (Fig. 5a–c). Thus MYC-induced DNA damage and apoptosis in response to PARPi was associated with transient activation of Chk1, suggesting that sustained ATR signaling contributes to PARPi resistance.

Consistent with Myc knockdown, which upregulated endogenous CDK18 (Fig. 2d), CDK18 overexpression in MGG4 increased olaparib-induced p-Chk1 by three-fold and abrogated the induction of c-PARP and γH2AX (Fig. 5d). Conversely, silencing of CDK18 in BT74 suppressed Chk1 activation and induced c-PARP and γH2AX upon olaparib treatment (Fig. 5d). CDK18 knockdown in the absence of Myc (MGG4-shMYC) also suppressed p-Chk1 and restored γH2AX and c-PARP levels (Fig. 5e), while CDK18 overexpression in the presence of Myc (BT74-MYC) restored the original phenotype: Chk1 activation and minimal γH2AX and c-PARP after olaparib (Fig. 5e). This demonstrates that CDK18, acting downstream of Myc, facilitates PARPi-induced ATR signaling and DNA repair.

### CDK18 interacts with ATR and promotes ATR activity

**Fig. 6:**
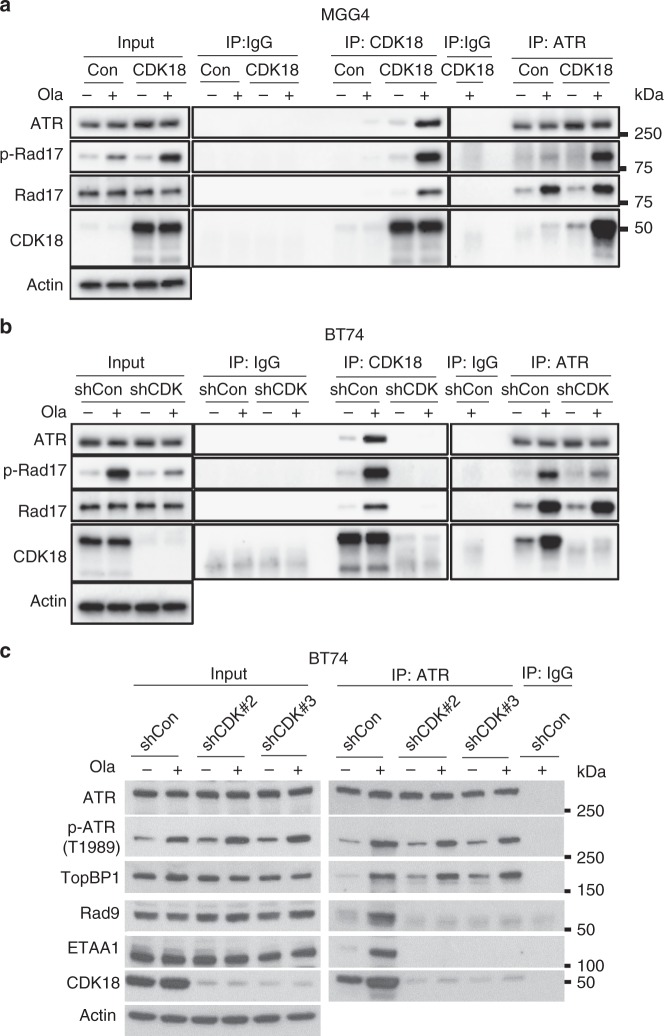
Cyclin-dependent kinase 18 (CDK18) interacts with ATR and regulates ATR–Rad9 and ATR–ETAA1 interactions. Immunoprecipitation (IP) with antibodies to IgG (control), CDK18, and ATR. **a** MGG4 with CDK18 or without (Con) and **b**, **c** BT74 with CDK18 knockdown (shCDK18^#2^ or as indicated) or not (shCon), treated with Ola (+; 10 μM) or mock (−) for 24 h. Doxycycline initiated 6 days before olaparib treatment. β-Actin was the loading control. Proteins are listed on the left, molecular weight markers are indicated to the right

Since CDK18 promoted Chk1 phosphorylation, indicative of ATR activity, we examined whether CDK18 physically interacted with ATR and other key components of ATR signaling complexes to activate ATR signaling triggered by PARPi. For this, we used CDK18-high BT74 cells with and without CDK18 knockdown (shCon, endogenous CDK18; shCDK) and CDK18-low MGGG4 cells with and without CDK18 overexpression (Con, endogenous CDK18; CDK18) (Fig. 6a–c). Olaparib increased auto-phosphorylation of ATR at Thr1989 (Fig. 6c), a very early event during ATR activation and TopBP1 independent, and phosphorylation of Rad17, an indicator of full activation of ATR (Fig. 6a, b). Immunoprecipitation (IP) of CDK18 or ATR showed that olaparib greatly increased protein–protein interactions between CDK18 and ATR (Fig. 6a, b). The ATR complex also included Rad17, which was increased by PARPi but was not altered by CDK18 (Fig. 6a, b). ATR is known to interact with the Rad17-RFC complex on damaged DNA, a critical event for ATR activation^28^. Co-IP of CDK18 with both ATR and Rad17 suggests that CDK18 interacts with active ATR signaling complexes at sites of DNA damage. Consistent with this, the ATR/CDK18 complexes are enriched for p-Rad17, a marker of ATR activation at sites of DNA damage^28^ (Fig. 6a, b). In the absence of CDK18, ATR complexes had similar levels of Rad17 but only minimal p-Rad17 (Fig. 6a, b).

There are two ATR signaling complexes containing TopBP1, activator of ATR, and ETAA1, a second activator of ATR, independent of TopBP1^28,29^. PARPi stimulated ATR interactions with TopBP1, ETAA1, and Rad9, a component of the Rad9-Rad1-Hus1 (9-1-1) complex that is recruited by Rad17 and then recruits/stimulates TopBP1 in the ATR-DNA complex (Fig. 6c). CDK18 knockdown abrogated binding of Rad9 and ETAA1 to ATR in response to PARPi but had no effect on TopBP1 or auto-phosphorylation of ATR at Thr1989 (Fig. 6c). Thus CDK18 participates in ATR complexes in response to PARPi-induced damage/replication stress and promotes the full activation of ATR, as indicated by p-Rad17 and p-Chk1, by regulating ATR–Rad9 and ATR–ETAA1 interactions.

### ATRi kills both non-Myc and Myc-amplified GSCs with PARPi

We demonstrated that CDK18 interacts with ATR and promotes PARPi-triggered activation (phosphorylation) of Chk1 and Rad17, two well-established ATR substrates serving as markers of ATR activity^30^, enhancing DNA repair and generating PARPi insensitivity in GSCs. This suggests that ATR, as a direct downstream effector of CDK18, is activated by PARPi and drives PARPi resistance in GSCs. High levels of CDK18 enable non-Myc GSCs to survive PARPi treatment by activating ATR. Therefore, we hypothesized that ATR blockade would kill not only Myc-amplified but also non-Myc GSCs in the presence of PARPi. We first evaluated selective ATRis VE822 (VX-970, M6620), AZ20, and VE821 as monotherapy. As expected, Myc-amplified GSCs (MGG4, MGG6 and MGG8) were more sensitive to ATRi than non-Myc GSCs (MGG18, BT74, and MGG24) (Supplementary Fig. 10a–c). Newer generation compounds, VE822 and AZ20, were more potent than VE821 (Supplementary Fig. 10a–c).

**Fig. 7:**
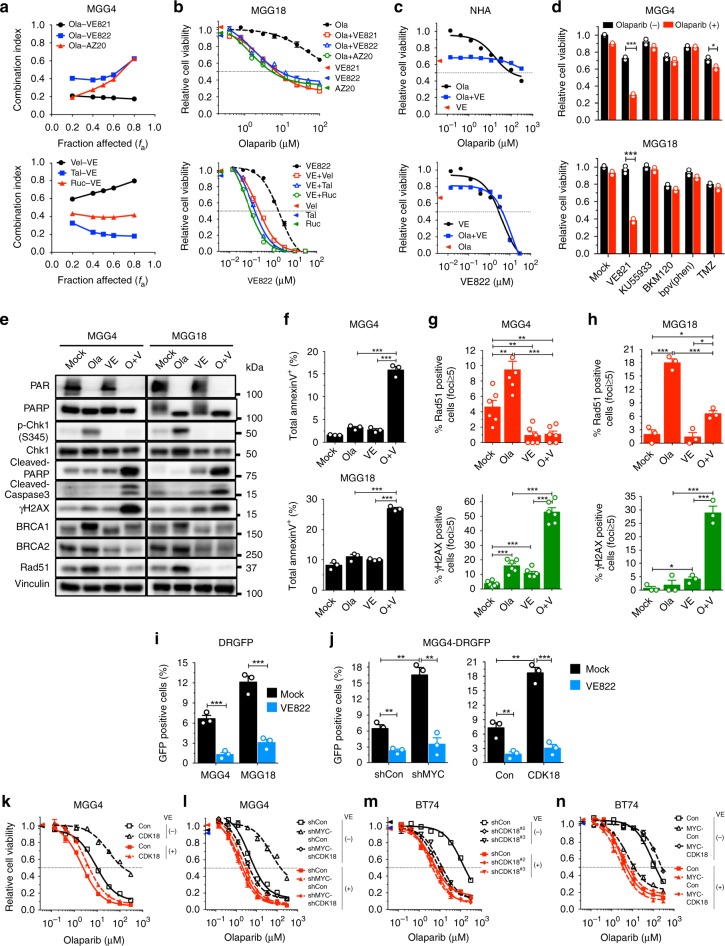
ATR inhibitor suppresses homologous recombination (HR) and synergizes with poly(ADP-ribose) polymerase inhibitor (PARPi). **a** Chou–Talalay analysis of MGG4 treated with Ola and different ATRis (upper) or VE822 (VE) with different PARPis (lower; veliparib (Vel), talazoparib (Tal), rucaparib (Ruc)). Combination Index <1 is synergistic. **b** Ola dose response in MGG18 with ATRis (10 μM VE821, 0.3 μM VE822, or 0.3 μM AZ20) (upper) or VE with 10 μM veliparib, 0.5 μM talazoparib, and 1 μM rucaparib (lower). **c** Ola dose response with VE (0.3 μM) (upper) or VE with Ola (10 μM) (lower) in normal human astrocytes (NHA). **d** Combination of olaparib (1, 10 μM) with VE821 (5, 10 μM), ATM inhibitor (KU55933; 5, 5 μM), PI3K inhibitor (BKM120; 0.2, 0.2 μM), PTEN inhibitor (bpV(phen); 4, 4 μM), and temozolomide (TMZ; 3, 10 μM) in MGG4 (upper) and MGG18 (lower). **e** Representative western blots (*n* = 3) of MGG4 and MGG18 treated with Ola (O; 10, 10 μM) and/or VE (V; 0.3, 3 μM) for 24 h. Vinculin was the loading control. **f** Annexin V assay (representative plots in Supplementary Fig. 12a) of MGG4 (upper) and MGG18 (lower) treated with Ola (O; 3, 10 μM), and/or VE (V; 0.1, 0.5 μM), for 72 h. **g**, **h** Quantification of RAD51 (upper) and γH2AX (lower) foci-positive MGG4 (**g**) and MGG18 (**h**) at 24 h after Ola (10, 10 μM) and/or VE (0.3, 3 μM). Representative images in Supplementary Fig. 12b. **i** HR quantification at 24 h after VE822 (0.1 μM for MGG4, 0.5 μM for MGG18). Representative plots in Supplementary Fig. 13a. **j** HR quantification at 24 h after VE822 (0.1 μM) in MGG4-DRGFP with shMYC#1 or not (shCon) (left), or cyclin-dependent kinase 18 (CDK18) overexpression or not (Con) (right). Representative plots in Supplementary Fig. 13b, c. **k**–**n** Olaparib dose response with (+) or without (−) VE (0.1 μM for MGG4 and 0.3 μM for BT74) in MGG4-CDK18 (**k**), MGG4-shMYC#1-shCDK18#2 (**l**), BT74-shCDK18 (**m**), and BT74-MYC-CDK18 (**n**). Cell viability measured by MTS assay after 6 days. Normalized to control and mean ± SEM, representative of three independent experiments performed in triplicate. *p < 0.05, **p < 0.01 and ***p < 0.001, *t* test. Colored triangles indicate viability for drug alone

We next assessed how ATRis and PARPi would interact for the treatment of Myc-amplified and non-Myc GSCs. All tested ATRis were synergistic with all tested PARPis in MYC-high GSC MGG4 (Fig. 7a). The sequence and timing of treatment did not alter the combination effect (Supplementary Fig. 11a). Olaparib and VE822 were also synergistic in two MYCN-amplified GSCs, MGG6 and MGG8 (Supplementary Fig. 11b). In non-Myc MGG18, one fixed non-toxic dose of ATRi (VE821, VE822, or AZ20) consistently sensitized cells to PARPis (olaparib, veliparib, talazoparib, or rucaparib) (Fig. 7b upper, Supplementary Fig. 11c), while one fixed non-toxic dose of PARPis consistently sensitized MGG18 to ATRis (Fig. 7b lower, Supplementary Fig. 11d). This combination effect was also observed in other non-Myc GSCs (BT74 and MGG24) (Supplementary Fig. 11e, f). Importantly, PARPi and ATRi did not increase cytotoxicity in normal human astrocytes (Fig. 7c). The interaction of PARPi and ATRi was specific; PARPi did not sensitize GSCs to other DDR inhibitors (ATMi (KU55933), PI3Ki (BKM120), PTENi (bpV(phen)) or temozolomide (TMZ) chemotherapy (Fig. 7d). Thus ATRi selectively synergizes with or sensitizes GSCs to PARPi, regardless of GSC sensitivity to PARPi.

### ATRi inhibits DDRs in combination with PARPi

To discern the mechanisms underlying combination therapy cytotoxicity, we evaluated how ATRi modifies DDR signaling. As expected, ATRi inhibited PARPi-induced Chk1 phosphorylation but not PARP activity in GSCs (PAR; Fig. 7e). In both Myc-amplified MGG4 and non-Myc MGG18 GSCs, combination therapy increased γH2AX and apoptosis markers (cleaved PARP, cleaved caspase 3, and Annexin V) (O+V; Fig. 7e, f, Supplementary Fig. 12a). Olaparib increased levels of key HR proteins, BRCA1, BRCA2, and Rad51, which was counteracted by VE822 (Fig. 7e), suggesting that ATRi suppresses HR in GSCs. Indeed, Rad51 foci formation, which marks HR repair of DSBs, was induced by olaparib but inhibited in combination with ATRi (Fig. 7g, h upper, Supplementary Fig. 12b), resulting in accumulation of γH2AX foci in both Myc-amplified and non-Myc GSCs (Fig. 7g, h lower, Supplementary Fig. 12b). Assaying for HR efficiency demonstrated that ATRi potently inhibited HR in both GSCs (Fig. 7i, Supplementary Fig. 13a).

Because of Myc/CDK18 effects on ATR signaling, we examined their effects on ATRi–PARPi interactions. ATRi blocked HR that was increased by MYC knockdown or CDK18 overexpression to levels seen in control MGG4 (Fig. 7j, Supplementary Fig. 13b, c). Hence, we investigated whether MYC or CDK18 expression in GSCs alters ATRi-mediated sensitization to PARPi. In MGG4, ATRi overrode PARPi insensitivity induced by CDK18 overexpression or MYC knockdown (Fig. 7k, l) and only modestly increased the sensitivity observed with double MYC and CDK18 knockdown (Fig. 7l). In non-Myc BT74, ATRi did not further enhance PARPi sensitivity after CDK18 knockdown (Fig. 7m**)**, slightly sensitized when only MYC was overexpressed (Fig. 7n), and reversed insensitivity when MYC and CDK18 were both expressed (Fig. 7n). These data collectively show that ATR promotes HR and PARPi resistance, as a downstream effector of CDK18.

### ATRi combined with PARPi improves antitumor efficacy

**Fig. 8:**
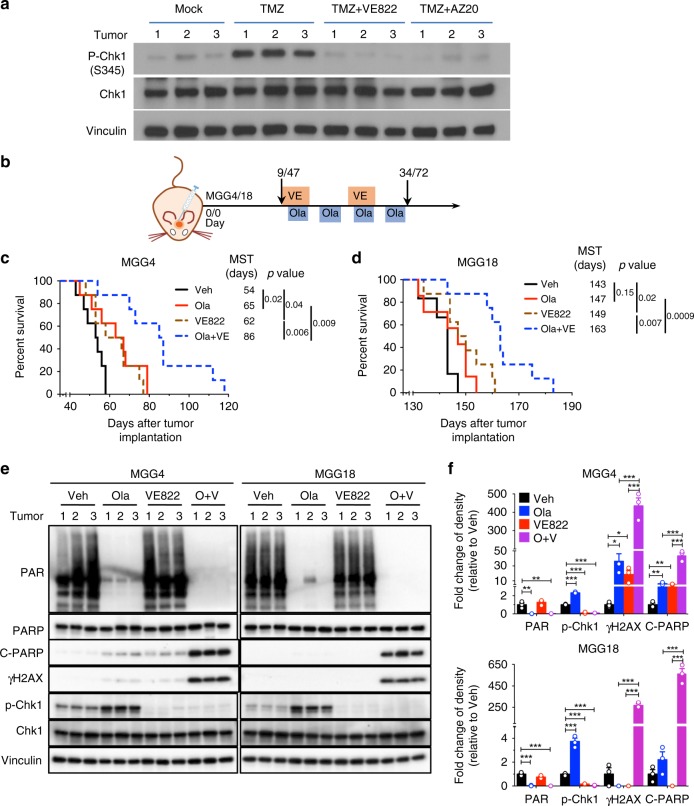
ATRi and poly(ADP-ribose) polymerase inhibitor combination is efficacious in glioblastoma stem-like cell (GSC)-derived intracerebral tumors. **a** Mice bearing intracerebral MGG4 GSC tumors at day 48 were treated with VE822 (60 mg/kg, gavage) or AZ20 (50 mg/kg, gavage) for 4 days. One hour after last dosing, mice were administered temozolomide (TMZ, 100 mg/kg, intraperitoneally (i.p.)), sacrificed 7 h later, tumor lysates prepared, and western blot analysis performed. Vinculin was the loading control. **b** Treatment schedule for **c**, **d**. VE822 (2 cycles, 60 mg/kg) from 1 day before Ola (4 cycles, 50 mg/kg, i.p.), with days listed for MGG4/MGG18, respectively. **c**, **d** Kaplan–Meier survival curves of mice bearing orthotopic MGG4 (**c**) or MGG18 (**d**) tumors treated as in **b**. MST median survival time. Vertical lines indicate *p* value comparisons (log-rank test). **e** Olaparib and VE822 induce DNA damage response in GSC tumors in vivo. MGG4 (left) and MGG18 (right) intracerebral tumors treated with vehicle (Veh), Ola (100 mg/kg), VE822 (60 mg/kg), or combination (O+V) for 6 consecutive days. Western blots from lysates from individual mouse tumors (1, 2, 3). Vinculin was the loading control. Proteins are listed on the left. **f** Quantification of PAR, p-Chk1, γH2AX, and cleaved-PARP (c-PARP) levels from western blots shown in **e**. Mean ± SEM (*n* = 3/group). *p < 0.05, **p < 0.01 and ***p < 0.001, *t* test

Finally, we assessed the efficacy of combination therapy in vivo in orthotopic brain tumors (Fig. 8). We previously showed that systemically administered olaparib was active in the brain^18^. However, it was unknown whether ATRi could cross the blood–brain/tumor barrier. To determine whether ATRi could inhibit ATR activity in brain tumors, we induced ATR signaling with TMZ after systemic administration of ATRi. Both VE822 and AZ20 inhibited Chk1 phosphorylation, indicative of ATR inhibition (Fig. 8a). In Myc-amplified MGG4 xenografts, olaparib (Ola) or VE822 alone modestly but significantly extended survival compared with vehicle (Fig. 8b, c). The combination of olaparib and VE822 further prolonged survival over control by >60% and was significantly better than either monotherapy (Fig. 8c). In non-Myc MGG18 xenografts, olaparib alone had no effect, while VE822 alone produced a modest prolongation in survival (Fig. 8b, d). However, the combination of olaparib and VE822 further extended survival (Fig. 8d). The treatments overall were well tolerated, causing no significant changes in body weight (Supplementary Fig. 14). Thus combination therapy safely improved efficacy in both Myc-amplified PARPi-sensitive and non-Myc PARPi-insensitive GSC-derived intracerebral tumor models.

### PARPi and ATRi induce DNA damage and apoptosis in vivo

To evaluate the in vivo effects of combination therapy on DDR, tumor lysates were analyzed by western blot. Olaparib potently inhibited PARP activity (Fig. 8e, f; PAR) and induced ATR signaling (p-Chk1) 2.3- and 3.7-fold in MGG4 and MGG18, respectively, which was totally blocked by ATRi (Fig. 8e, f; p-Chk1). DNA damage and apoptosis were modestly induced by PARPi or ATRi alone in MGG4 and greatly increased by the combination (Fig. 8e, f; γH2AX and c-PARP). In non-Myc MGG18, only combination treatment significantly induced DNA damage and apoptosis (Fig. 8e, f). These in vivo data are consistent with the in vitro results and indicate that reduced DNA damage repair and increased apoptosis underlie efficacy.

## Discussion

**Fig. 9:**
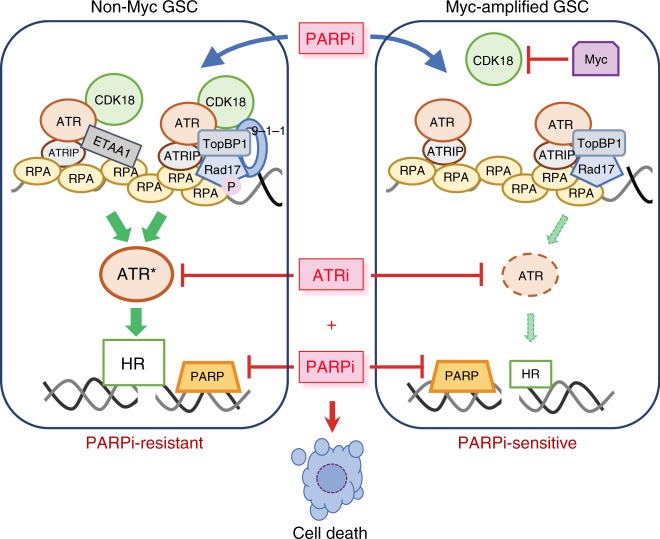
Model for Myc/cyclin-dependent kinase 18 (CDK18) regulation of ATR signaling and poly(ADP-ribose) polymerase inhibitor (PARPi) sensitivity in glioblastoma stem-like cells (GSCs). In non-Myc GSCs (left), CDK18 fully activates ATR via ATR-ETAA1 and ATR-TopBP1 complexes containing Rad9 (9-1-1) and Rad17, which increases homologous recombination (HR), and generates PARPi resistance. In Myc-amplified GSCs (right), Myc amplification/overexpression inhibits CDK18 expression so that CDK18 interactions with ATR signaling complexes after PARPi-induced DNA damage are lacking, leading to suppressed ATR signaling and HR, which generates PARPi sensitivity. ATRi sensitizes both non-Myc GSCs (PARPi-resistant; left) and Myc overexpressing GSCs (PARPi-sensitive; right) to PARPi killing (cell death)

GSCs established from different patients exhibited differential sensitivity to PARPis. We discovered that overexpression of MYC/MYCN induces PARPi sensitivity through direct transcriptional repression of CDK18 alone. CDK18, a poorly understood CDK, interacts with ATR after PARPi treatment and facilitates ATR signaling and HR repair, recapitulating the effects of low Myc on DDR. Therapeutically, ATRi enhances PARPi sensitivity in Myc-amplified/overexpressing GSCs and overcomes PARPi resistance in non-Myc GSCs in vitro and in vivo (Fig. 9).

Efforts to expand the use of PARPis beyond cancers with BRCAness have identified cancer-associated molecular alterations other than *BRCA1/2* that generate PARPi sensitivity in cancer^4,31–34^. In GBM, where *BRCA1/2* functional mutations/deletions are rare (≤1%) (cBioPortal)^35,36^, 20% of tested GSCs were reported to respond to PARPi veliparib^17^. We found an association between *MYC* and *MYCN* amplification and PARPi sensitivity in our panel of human GSCs. A key oncogene deregulated in many cancer types, *MYC/MYCN* contributes to a broad array of tumorigenic phenotypes^24^, including cancer cell proliferation, metabolism, and stemness^22,37^. Therefore, it was important to manipulate Myc expression transiently. Myc knockdown in MYC-amplified GSCs after tumor initiation impaired tumor growth, confirming a role for Myc in GBM maintenance/progression^37^. There has been only limited study of the effect of Myc amplification/overexpression on PARPi sensitivity. In triple-negative breast cancer, where *MYC* is frequently amplified, especially in BRCA mutant tumors, the CDK inhibitor dinaciclib downregulated MYC as well as HR genes and sensitized PARPi-resistant cells to PARPi^38^, opposite to our Myc results in GSCs. A clinical trial of dinaciclib in combination with PARPi veliparib in solid tumors was poorly tolerated, with limited efficacy^39^. On the other hand, *MYC* amplification and genomic rearrangements strongly correlated with PARPi sensitivity in ovarian cancer cell lines^40^.

We show that direct transcriptional repression of CDK18 by Myc suppresses HR and mediates sensitization to PARPis in GSCs. Both MYC and MYCN bind the promoter of CDK18 at the CCCTCCC motif that is enriched in Myc-repressed promoters^25^. Myc represses many genes that encode negative regulators of cell cycle progression: e.g., *CDKN2B* (p15ink4b), *CDKN1A* (p21cip1), and *CDKN1B* (p27kip1)^41–43^, while activating transcription of many positive cell proliferation regulators. In line with this, we also found Myc repression of *CDKN1A* and activation of *CCND2*, encoding cyclin D2, in MYC-amplified GSCs. However, in contrast to other cancers, we did not observe MYC-mediated transcriptional activation of DDR genes such as *CHK1*, *PARP1*, *PARP2*, and *BRCA1*^44–46^.

CDKs play vital roles in cell cycle regulation and HR repair^47^, and inhibition of CDK1 or 12 sensitizes cancer cells to PARPis^48,49^. CDK18 (PCTK3) is a relatively uncharacterized CDK of unknown physiological function that belongs to the PCTAIRE subfamily of CDKs, which includes CDK16 and CDK17^50^. CDK18 is amplified in about 20% of invasive breast carcinomas, 12% of metastatic prostate cancers, and 5% serous ovarian cancers (cBioPortal), suggesting its role as a cancer driver. GSCs with inducible MYC/MYCN/CDK18 overexpression or silencing allowed us to define the critical role that CDK18 plays in regulating GSC responses to PARPis. CDK18 deficiency impaired HR and was sufficient and necessary for PARPi killing of GSCs, thus identifying a synthetic lethal interaction. We suggest that CDK18 promotes HR by enhancing the localization of BRCA2 to DSBs, which is an ATR-regulated process^51^. The dominance of CDK18 in determining PARPi sensitivity was unexpected given the myriad genes and signaling pathways impacted by Myc overexpression^24,25^. GSCs with low MYC or high CDK18 responded to PARPi with higher, stable levels of p-Chk1, a key substrate of ATR^30^. Recently, CDK18 was reported to support genome stability: promoting S-phase transit and ATR signaling in response to replication stress, through its interactions with Rad9, Rad17, and TopBP1^52^. We showed that PARPi-induced DNA damage triggered ATR activation that proceeds through ATR signaling complexes regulated by CDK18. We identified a function of CDK18: promoting the full activation of ATR in the presence of PARPi, as denoted by greatly increased p-Rad17 in the complex, via regulating the interactions of ATR with Rad9 and ETAA1 (Fig. 9). Our data support a key role of CDK18 in both TopBP1-Rad9-driven and ETAA1-driven ATR activation^29^. We thus identify Myc expression/CDK18 repression as an additional pathway for “BRCAness” that offers a avenue for PARPi therapy and propose CDK18 as an important target for drug development.

The current work addresses the challenge associated with therapeutic targeting of molecularly heterogeneous tumor cells. ATRis potently inhibited HR and broadly increased PARPi sensitivity in both PARPi-sensitive Myc-amplified and PARPi-insensitive non-Myc GSCs. ATRi was dominant, overriding the PARPi-insensitive phenotype induced by Myc knockdown or CDK18 overexpression, indicating that ATR functions as a direct downstream effector of CDK18 in HR and DDR. Previously, cancer cells that express low-level Myc were considered independent of ATR, whereas Myc-driven cancer cells were characterized by increased replication stress and dependence on ATR to maintain genome stability^53,54^. ATRis have been shown to sensitize BRCA1/2-deficient and wild-type ovarian and breast cancer cell lines^55–57^ and sarcoma cells^58^ to PARPis, leading to a number of clinical trials in patients with solid tumors (i.e., NCT03462342, NCT03682289, NCT02723864). Recently, it was shown that GSCs, but not matched bulk serum-cultured cells, were sensitive to the combination of ATRi and PARPi in vitro at doses that were non-toxic for each agent alone^59^. Here we demonstrate the efficacy of ATRi and PARPi combination therapy for *BRCA1/2* wild-type tumors in vitro and in vivo. ATRi VE822 had the ability to penetrate the blood–brain/tumor barrier and, when combined with olaparib in vivo, abrogated PARPi-induced p-Chk1, increased apoptosis and DNA damage, and extended animal survival in orthotopic GBM models generated from either *MYC*-amplified or non-Myc GSCs. The combination treatment was safe in mice and did not increase toxicity to normal astrocytes in vitro. The exact mechanisms underlying PARPi–ATRi combination selectivity in cancer but not in normal cells remains to be investigated.

A major finding of our studies is that the CDK18-ATR signaling axis regulates HR and consequently PARPi sensitivity in GBM (Fig. 9). We provide a mechanistic model whereby *MYC/MYCN* amplification induces PARPi sensitivity in GSCs by binding to the CDK18 promoter and repressing CDK18 expression. CDK18 is an interactor with ATR that promotes ATR activation and stimulates HR, enhancing the localization of BRCA2 to DSBs, which facilitates Rad51 foci formation in S/G2-phase cells, thereby suppressing PARPi sensitivity. Combined blockade of ATR and PARP is an effective therapeutic strategy for GBM, even in non-Myc PARPi-resistant GSCs, thus providing a much-needed therapeutic breakthrough for this deadly disease and possibly other *BRCA1/2*-wild-type or HR proficient cancers. Furthermore, MYC/MYCN and CDK18 may offer predictive biomarkers for PARPi responsiveness. Development of pharmacological inhibitors of CDK18 should afford cancer therapeutics that would enhance the efficacy of PARPi and DNA-damaging agents. Our work has a number of clinically relevant implications: *MYC/MYCN*-amplified GBM, although in a minority of patients, and possibly other *MYC/MYCN*-amplified brain tumors (e.g., medulloblastoma), should be sensitive to PARPi alone; the combination of ATRi and PARPi does not depend on PARPi sensitivity; and the combination of ATRi and PARPi should be clinically evaluated for GBM, even in the absence of radiation and/or TMZ, such as in recurrent GBM for which there are no current therapies.

## Methods

### Cells

Human GSCs (MGG4, MGG6, MGG8, MGG152, MGG13, MGG18, MGG24), isolated from dissociated surgical tumor specimens^10,13,21^ (with approval of MGH Institutional Review Board), and BT74, isolated from patient-derived xenograft GBM6^60^ (obtained from Dr S. Kesari, Brigham and Women’s Hospital, Boston, MA) were cultured in EF20 medium composed of Neurobasal medium (ThermoFisher Gibco) supplemented with 3 mM L-Glutamine (Corning Mediatech), 1 × B27 supplement (ThermoFisher Gibco), 0.5 × N2 supplement (ThermoFisher Gibco), 2 μg/ml heparin (Sigma, St Louis, MO), 20 ng/ml recombinant human epidermal growth factor (R&D Systems, Minneapolis, MN), 20 ng/ml recombinant human fibroblast growth factor-2 (PeproTech, Rocky Hill, NJ), and 0.5 × penicillin G/streptomycin sulfate/amphotericin B complex (Corning Mediatech) at 37 °C and 5% CO2. To passage cells, neurospheres were dissociated with the NeuroCult Chemical Dissociation Kit (StemCell Technologies). These GSCs (not all for each feature) have been characterized by us and other investigators for sphere-forming ability and tumorigenicity^10,13,14,21,61^, epigenetic and genetic alterations^10,14,21,61,62^, gene expression and subtype classification^14,61,62^, and in vitro self-renewal^13,14,61,63^ and differentiation^13,14,62^. ScGCs from the same tumor specimens as GSCs were isolated as described^13^, and grown in Dulbecco’s modified Eagle’s medium (DMEM) supplemented with 10% fetal calf serum (FCS). 293T cells, obtained from American Type Culture Collection (ATCC, Manassas, VA), and normal human astrocytes from ScienCell (Carlsbad, CA) were cultured in complete DMEM supplemented with 10% FCS. Cells were confirmed to be mycoplasma-free (LookOut Mycoplasma kit, Sigma) and used at low passage number.

### Chemotherapeutic drugs and compounds

Olaparib (MedChem Express), veliparib (ABT888; Selleck), rucaparib (AG014699; Selleck), talazoparib (BMN673; Selleck), VE822 (MedChem Express), AZ20 (MedChem Express), VE821 (Selleck), KU55933 (Selleck), BKM120 (Selleck), and TMZ (Sigma) were dissolved in dimethyl sulfoxide (DMSO). bpV(phen) (Millipore) was dissolved in water for stock. Chemicals were diluted with medium for in vitro studies.

### Inducible knockdown of MYC, MYCN, or/and CDK18

shRNA oligonucleotides against MYC, MYCN, CDK18, or scrambled shRNA (control or Con in figures; sequences in Supplementary Table 3) were synthesized (Thermo Fisher Scientific) and cloned into inducible lentivector Tet-pLKO-puro (Addgene) according to the manufacturer’s instructions. 293T cells were transfected with packaging plasmid (psPAX2), envelope plasmid (pCMV-VSV-G), and shRNA-cloned Tet-pLKO-puro using FuGENE HD Transfection Reagent (Promega). Lentivirus was harvested at 48 h after transfection and used to infect GSCs, followed by selection with puromycin (0.1–0.6 μg/ml, Sigma) for 7–10 days. Cells were induced with Dox (Sigma; 1 μg/ml) for 4 days and protein levels/silencing were assessed by western blot analysis. Cells were induced for 6 days prior to use in experiments.

### Inducible expression of MYC, MYCN, or/and CDK18

PCDH-puro-cMyc (Addgene), pDNR-Dual-MYCN (PlasmID; Dana-Farber Harvard Cancer Center DNA Resource Core), and pDONR223-PCTK3 (Addgene) were used for PCR amplification of human MYC, MYCN, and CDK18 cDNAs, respectively (primer sequences in Supplementary Table 4), which were cloned into inducible lentiviral expression vector pINDUCER21 (Addgene). Control is empty vector (no cDNA). Lentiviruses were packaged as described above. Transduced GSCs were sorted by fluorescence-activated cell sorter for EGFP expression and Dox induced (1 μg/ml) for 4 days to assess protein expression by western blot. Cells were induced for 6 days prior to use in experiments.

### Cell cycle analysis

Cells were plated in triplicate in 6-well plates at a density of 5 × 10^5^ cells/well overnight and labeled with EdU (10 μM) for 30 min using the Click-iT® Plus EdU Flow Cytometry Assay Kit (Invitrogen) according to manufacturer’s instruction. Before flow cytometry, cells were stained in propidium iodide (PI, 50 μg/ml) with 100 μg/ml RNase at 37 °C for 20 min.

### Cell growth assay

Cell were plated at 2 × 10^4^ cells/well, fed once at 3 days after plating, and harvested for counting using trypan blue exclusion assay at 3 and 7 days after plating.

### Cell viability assay

Dissociated cells were plated in 96-well plates and treated the following day. Cell viability was measured 6 days after treatment by MTS (3-(4,5-dimethylthiazol-2-yl)-5-(3-carboxymethoxyphenyl)-2-(4-sulfophenyl)-2H-tetrazolium) assay (Promega), according to the manufacturer’s instructions. Results were analyzed in the Prism GraphPad software and IC_50_ was calculated from the dose–response curve (nonlinear curve fit).

### Chou–Talalay assay

Cells were treated with three-fold serial dilutions of PARPi and ATRi alone or in combination in a ratio equal to the ratio of their IC_50_ values for 6 days followed by MTS assay. The median-effect dose (D_*m*_) was obtained from the dose–response curves according to the equation log (fa/fu) = *m*logD − *m*logD_*m*_, where fa is the fraction affected, fu is the fraction unaffected, *D* is the dose, and *m* is the coefficient signifying the shape of the dose–response curve^64^. The combination indices (CIs) were calculated by the equation CI = (*D*_1_/Dx_1_) + (*D*_2_/Dx_2_) + (*D*_1_) (*D*_2_)/[(Dx_1_)(Dx_2_)], in which Dx_1_ and Dx_2_ are the doses of PARPi and ATRi, respectively, required to obtain a particular fa, and *D*_1_ and *D*_2_ the combination doses required for the same fa. CI <1, =1, and >1 indicate synergistic, additive, and antagonistic interactions, respectively.

### Western blotting

Protein (10–20 μg/lane) was separated by 4–15% sodium dodecyl sulfate-polyacrylamide gel electrophoresis (BioRad) and transferred to polyvinylidene difluoride membranes (Bio-Rad) by electroblotting. Membranes were blocked with 5% nonfat dry milk for 1 h at room temperature and then incubated with primary antibodies (Supplementary Table 5) at 4 °C overnight. Membranes were then washed in TBST (20 mM Tris pH7.5, 150 mM NaCl, 0.1% Tween20) and incubated with appropriate peroxidase-conjugated secondary antibodies (Promega and Cell Signaling Technology) for 1 h at room temperature. Signals were visualized with an ECL Kit (Amersham Bioscience or BioRad). Band intensities were quantified using Image Lab (Bio-Rad).

### RNA sequencing and pathway analyses

Cells (1 × 10^6^) were harvested and RNA extraction performed with Trizol (Invitrogen) according to the manufacturer’s instructions. Total RNA (1 μg) underwent two rounds of mRNA purification (polyA-selection) using the Dynabeads mRNA DIRECT Kit (Invitrogen, Carlsbad, CA). Double-stranded cDNA was generated using the Superscript III First-Strand Synthesis System (Invitrogen, Carlsbad, CA). The cDNA products were used to construct libraries with the Nextera XT DNA Sample Preparation Kit (Illumina, Inc., San Diego, CA). Libraries were sequenced on the Illumina NextSeq 500. Reads were aligned to the hg19 human genome using Spliced Transcripts Alignment to a Reference (STAR)^65^. Data are presented as the number of reads per kilobase of transcript per million total reads^66^.

To analyze the enrichment of pathways, genes with logFC > 0 and *p* value < 0.05 were considered significantly upregulated, and genes with logFC < 0 and *p* value < 0.05 were considered significantly downregulated. For each of the categories, gene ontology (GO) analyses was performed using DAVID version 6.8^67,68^, and GO_Term_BP_Direct Gene Ontology category, and results were output as Functional Annotation Charts. These results were used as input for the Enrichment Map tool^69^ for Cytoscape version 3.4.0^70^, with the following parameters: *p* value cutoff = 0.005; false discovery rate *Q*-value cutoff = 0.1; overlap coefficient cutoff = 0.5. GO terms representing similar biological processes were grouped into clusters.

### Quantitative RT-PCR

Cells with MYC knockdown (shRNA), MYC/MYCN overexpression, or control cells were induced with Dox (1 μg/ml) for 6 days and harvested for RNA extraction with Trizol (Invitrogen) according to the manufacturer’s protocol. CDNA reaction was done using the High-Capacity cDNA Reverse Transcription Kit (Applied Biosystems). Quantitative PCR was performed with SYBR green PCR master mix (Applied Biosystems) in a real-time PCR machine (Step One Plus Real-Time PCR System, Applied Biosystems).

### ChIP and PCR

Cells were dissociated and cultured in fresh media for 3 days before harvested for ChIP using a Chromatin Immunoprecipitation Kit (EZ-Magna ChIP A, Millipore), according to the manufacturer’s Instruction Manual. Briefly, cells were fixated with 1% formaldehyde and lysed, followed by sonication using a Qsonica sonicater. Magnetic beads and antibodies for MYC (Cell Signaling), MYCN (Santa Cruz), or IgG (Cell Signaling and Santa Cruz) were incubated with the chromatin at 4 °C overnight. Input chromatin was used as control. Precipitated material was eluted and the crosslink was reversed. Purified DNA was amplified with PCR using primers for Myc-binding site (P1: left 5′ TGGTCAGTAAGATTTATTGGCTGT 3′ and right 5′ CAGGGAGGGTGCCAGAAC 3′) and non-Myc-binding site (P2, left 5′ AAGTGGAGGGGAGGTGAGAC 3′ and right 5′ CTGGCCACCCAATGAGAC 3′). MGG4 DNA was used as template for PCR as a positive control.

### Immunoprecipitation

IPs were performed on GSCs with lentivirus-mediated inducible CDK18 overexpression or knockdown. Cells were harvested at 48 h after olaparib (10 μM) treatment and lysed in lysis buffer (Cell Signaling). After pre-clearing, cell lysates were added to primary antibodies to CDK18 (Santa Cruz) or ATR (Bethyl) and incubated with rotation overnight at 4 °C. Next day, protein A agarose beads (10–30 µl of 50% bead slurry) were added, followed by incubation with rotation for 2 h at 4 °C. After washing with lysis buffer, proteins were analyzed by western immunoblotting.

### Construction of promoter-Luc reporter

An alternative promoter was predicted upstream of exon2 of *CDK18* by analyzing the genomic sequence using the online software FPROM. The predicted promoter for CDK18 (P_CDK_) was amplified by PCR with primers (left 5′ CTTTTAATTAATTCTTGAGAATGGGGACCAC 3′ and right 5′ CTTAGATCTCCTGTATGCCACCATCACTG 3′) and cloned into lentiviral vector plasmid pLN 411, which contains a luciferase gene under human ubiquitin C promoter (P_UbC_) (kindly provided by Dr. Lior Nissim at Massachusetts Institute of Technology, Cambridge). A mutant CDK18 promoter with deletion of Myc-binding site (CCCTCCC) (P_CDKΔ_) was generated by PCR with primer 1 (left 5′ CTTTTAATTAATTCTTGAGAATGGGGACCAC 3′ and right 5′ GTGGAATTCCAGAACCAGGCAGT 3′) and primer 2 (left 5′ TTCTGGAATTCCACCCCAGCCCTTC 3′ and right 5′ CTTAGATCTCCTGTATGCCACCATCACTG 3′), and cloned into pLN411 to replace P_UbC_, called pLN-P_CDKΔ._ Lentiviruses were packaged as above and GSCs with MYC/MYCN knockdown or overexpression infected to express luciferase driven by P_UbC_, P_CDK_, or P_CDKΔ_, respectively. Luminescence assay was performed 7 days after infection.

### HR assay

GSCs were transduced with plasmid pDRGFP (Addgene) by electroporation (230 V, 950 μF) and DRGFP+ cells were selected in puromycin (0.3–0.6 μg/ml) for 14 days. Transfection was confirmed by PCR with primers specific for DRGFP (5′ AGGGCGGGGTTCGGCTTCTGG 3′ and 5′ CCTTCGGGCATGGCGGACTTGA 3′). For knockdown or overexpression of MYC, MYCN, or/and CDK18, GSC-DRGFP cells were infected with lentiviruses described above and protein levels were assessed by western blot. Cells were induced with Dox (1 μg/ml) for 6 days before infection with lentivirus expressing I-SceI or no transgene. To construct the I-SceI lentivirus, I-SceI cDNA was cut (XbaI - BglII) from pCBASceI (Addgene) and inserted into lentivector pCDH-CMV (Addgene) (XbaI - BamHI) to generate pCDH-CMV-SceI. GSC-DRGFP cells were infected with lentivirus expressing I-SceI (I-SceI (+)) or expressing no transgene (I-SceI (−)) packaged as described above. At 5 days after infection, the percentage of GFP+ cells were analyzed by flow cytometry. For the effect of ATRi on HR, cells were treated with ATRi at 24 h before flow cytometry.

### Annexin V staining

Cells (4 × 10^5^) were plated, treated the next day with PARPi and ATRi for 3 days, then collected and washed in cold phosphate-buffered saline (PBS) for Annexin V and PI staining with the Annexin V Apoptosis Detection Kit APC (ThermoFisher eBioscience) according to the manufacturer’s protocol. Cells were analyzed by flow cytometry.

### Immunofluorescence staining

Cells (4 × 10^5^) were plated, treated with PARPi and ATRi for 12 or 24 h, washed in cold PBS, and cytospun onto a glass slide at 79 × *g* for 5 min. Cells were treated with 0.5% Triton and fixed in cold methanol, followed by blocking in PBS with 0.1% Triton, 2% bovine serum albumin and 10% milk. Cells were incubated with primary anti-γH2AX antibody (1:500, Millipore), anti-BRCA2 (1:1000, Millipore), anti-Rad51 antibody (1:200, Santa Cruz), and anti-RPA (1:200, Thermo Fisher Scientific) overnight at 4 °C in a humidified chamber after incubation with primary anti-γH2AX antibody (1:500, Millipore) for 2 h at room temperature for double staining. Following washing with TBST (Boston Bioproduct), cells were incubated with secondary antibodies Alexa-488 anti-mouse (1:250; Jackson ImmunoResearch) and Cy3 anti-rabbit (1:250; Jackson ImmunoResearch) for 1 h at room temperature, and slides were mounted with VectaShield (DAPI included, Vector Laboratories). Staining was imaged at ×60 with a Nikon 90i microscope and quantified using the software ImageJ.

### In vivo experiments

Dissociated GSCs were stereotactically implanted intracerebrally (right striatum, 2.5-mm lateral from Bregma and 2.5-mm deep) in 7–8-week-old female mice (SCID for MGG18 and athymic for other GSCs; National Cancer Institute, Frederick, MD)^13^ and treated as shown in Table 1. There were 6–8 mice/group for survival experiments and 3 mice/group for protein analysis experiments.

**Table 1:** Animal experiments

GSCs	Implanted cell number/mouse	Days after implantation to start treatment					
		Survival			Western blot		
		Ola	VE822	Dox	Ola	VE822	Dox
MGG4-shMYC	2 × 10^5^	12	—	9	—	—	34
BT74-MYC	1 × 10^5^	11	—	8	—	—	33
MGG4-CDK18	2 × 10^5^	8	—	5	—	—	—
BT74-shCDK18	2 × 10^5^	7	—	4	—	—	—
MGG4	2 × 10^5^	10	9	—	35	35	—
MGG18	2 × 10^5^	48	47	—	120	120	—

*GSC* glioblastoma stem-like cell, *Ola* olaparib, *Dox* doxycycline

To examine the effect of MYC (implantation with MGG4-shMYC or BT74-MYC) or CDK18 (implantation with MGG4-CDK18 or BT74-shCDK18) status on PARPi response in vivo, olaparib, 50 mg/kg in 10% DMSO/10% 2-hydroxyl-propyl-β-cyclodextrine/PBS, or vehicle was administrated intraperitoneally (i.p.) with 4 cycles of 5-day on and 2-day off dosing and with or without Dox (1 mg/ml, 31 days in drinking water with 2% sucrose) starting at the indicated days after implantation (Table 1). To examine the effect of PARPi and ATRi in MYC (MGG4) or non-MYC (MGG18) GSC-derived tumor models, olaparib was administrated as above, and VE822 (60 mg/kg) in 10% DMSO/40% propylene glycol/50% water or vehicle was orally delivered by gavage for 2 cycles of 6-day on and 8-day off dosing starting at the indicated days after implantation following Table 1. Animals were monitored for clinical symptoms, moribund mice were sacrificed and the presence of tumor was confirmed. Animal facility staff that monitored symptoms were blinded to treatments.

To confirm in vivo MYC knockdown and overexpression, mice implanted with MGG4-shMYC or BT74-MYC were treated with Dox (1 mg/ml) in drinking water with 2% sucrose or vehicle for 10 days before harvesting brain tumors. To assess ATRi activity in vivo, mice implanted with MGG4 GSCs were treated with VE822 (60 mg/kg, gavage) or AZ20 (50 mg/kg, gavage) from day 48 post-implantation for 4 days. One hour after the last dosing, mice were administered TMZ (100 mg/kg, i.p.) and sacrificed after 7 h. To examine DDR induced by olaparib and VE822, mice implanted with MGG4 or MGG18 were treated with olaparib (100 mg/kg, i.p), VE822 (60 mg/kg, gavage), or vehicle for 6 days. Mice were sacrificed at 5 h after the last treatment of olaparib and VE822 and tumors were removed. Tumors were frozen on dry ice, thawed, and homogenized in RIPA buffer with protease and phosphatase inhibitors, followed by western blot analysis. All in vivo procedures were approved by the Institutional Animal Care and Use Committee (IACUC) at Massachusetts General Hospital (Boston, MA).

### Statistical analysis

Chi-square test (2 × 2 test) was used to analyze RNA sequencing data and log-rank (Mantel–Cox) test for in vivo survival. Bivariate correlation analysis (Pearson’s *r* test) was used to examine the correlation of two variables in specimens from GBM patients. The data met the assumptions of tests. All other experimental results were analyzed using unpaired two-sided Student’s *t* test or Kaplan–Meier plot and log-rank (Mantel–Cox) test for survival, as indicated in figure legends (Prism; GraphPad). *p* < 0.05 was considered statistically significant.

### Reporting summary

Further information on research design is available in the Nature Research Reporting Summary linked to this article.

## Supplementary information

Supplementary Information

Reporting Summary

Source Data

## Data Availability

The authors declare that all data supporting the findings of this study are available within the article and its Supplementary Information Files or from the corresponding author upon reasonable request. Raw data files for RNAseq have been deposited in the NCBI Gene Expression Omnibus database under the accession code GSE131218. Full western blots (underlying Figs. 1, 2, 3, 5, 6, 7, 8) are presented in source data file.
