# Supplementary material for: Myc targeted CDK18 promotes ATR and homologous recombination to mediate PARP inhibitor resistance in glioblastoma
Source: Nat Commun. 2019 Jul 2;10:2910. doi: 10.1038/s41467-019-10993-5 (PMC6606647; doi:10.1038/s41467-019-10993-5)
Supplement: Supplementary file 1 — Supplementary Information [file 41467_2019_10993_MOESM1_ESM.pdf]

Supplementary Information

Ning J. et al.

Myc targeted CDK18 promotes ATR and homologous recombination to mediate PARP inhibitor resistance in glioblastoma

Supplementary Figures 1-14 and associated legends.

Supplementary Tables 1-5.

Supplementary Figure 1

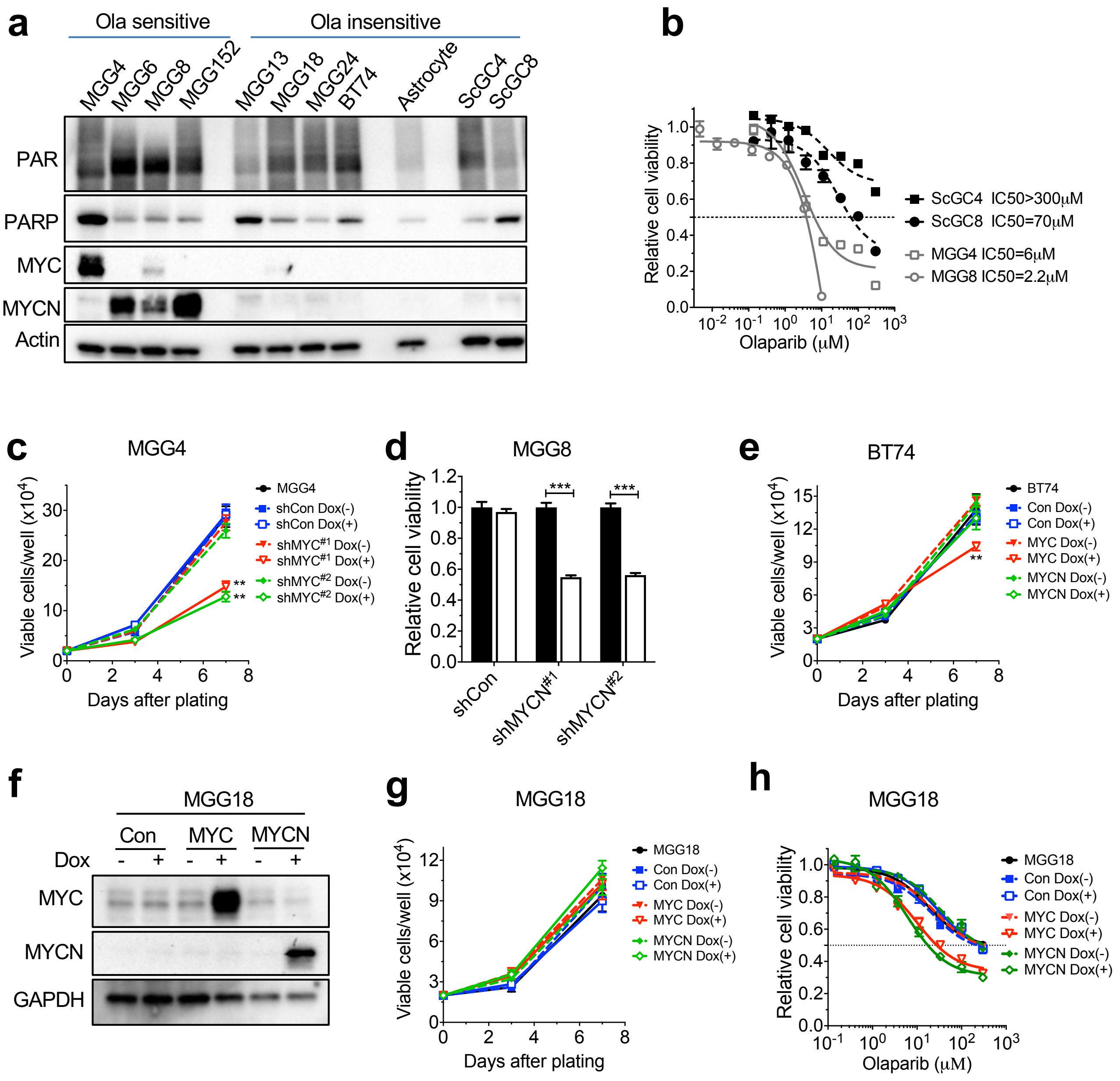

Supplementary Figure 1. MYC/MYCN overexpression induces GSC sensitivity to PARPis.

a. Western blot showing expression of PARP, parylated products (PAR), MYC and MYCN in PARPi-sensitive and -insensitive GSCs and ScGCs (serum cultured GBM cells). β-actin was the loading control.

b. Cell viability assay on indicated GSCs and ScGCs treated with PARPi. Cells treated with olaparib for 4 days, followed by MTS assay.

c. Viable cell counts (trypan blue exclusion, in triplicate) showing anti-proliferative effect of MYC knockdown on MGG4 growth. Control shRNA (shCon) or MYC shRNA (2 separate sequences; same as Fig. 1) transduced MGG4 cells were counted on days 3 and 7 with (+) or without (-) doxycycline (Dox, 1 μg/ml).

Supplementary Figure 1. ***Continued***

d. Effect of MYCN knockdown (shMYCN; 2 separate sequences; same as Fig. 1) in MGG8 on cell viability (MTS assay; triplicate) on day 6 under doxycycline.

e. Viable cell counts (in triplicate) after MYC or MYCN overexpression in BT74 with (+) or without (-) Dox.

f. Western blot of Dox-inducible overexpression of MYC or MYCN in MGG18 on day 4. GAPDH was loading control.

g. Viable cell counts (in triplicate) after MYC or MYCN overexpression in MGG18.

h. MYC/MYCN overexpression increases PARPi sensitivity. Olaparib dose response (MTS assay) in MGG18-MYC or -MYCN cells with (+) or without (-) Dox for 6 days.

Data are normalized to control cells and mean ± SEM from a representative experiment (n=3). ***p<0.001, t-test.

Supplementary Figure 2

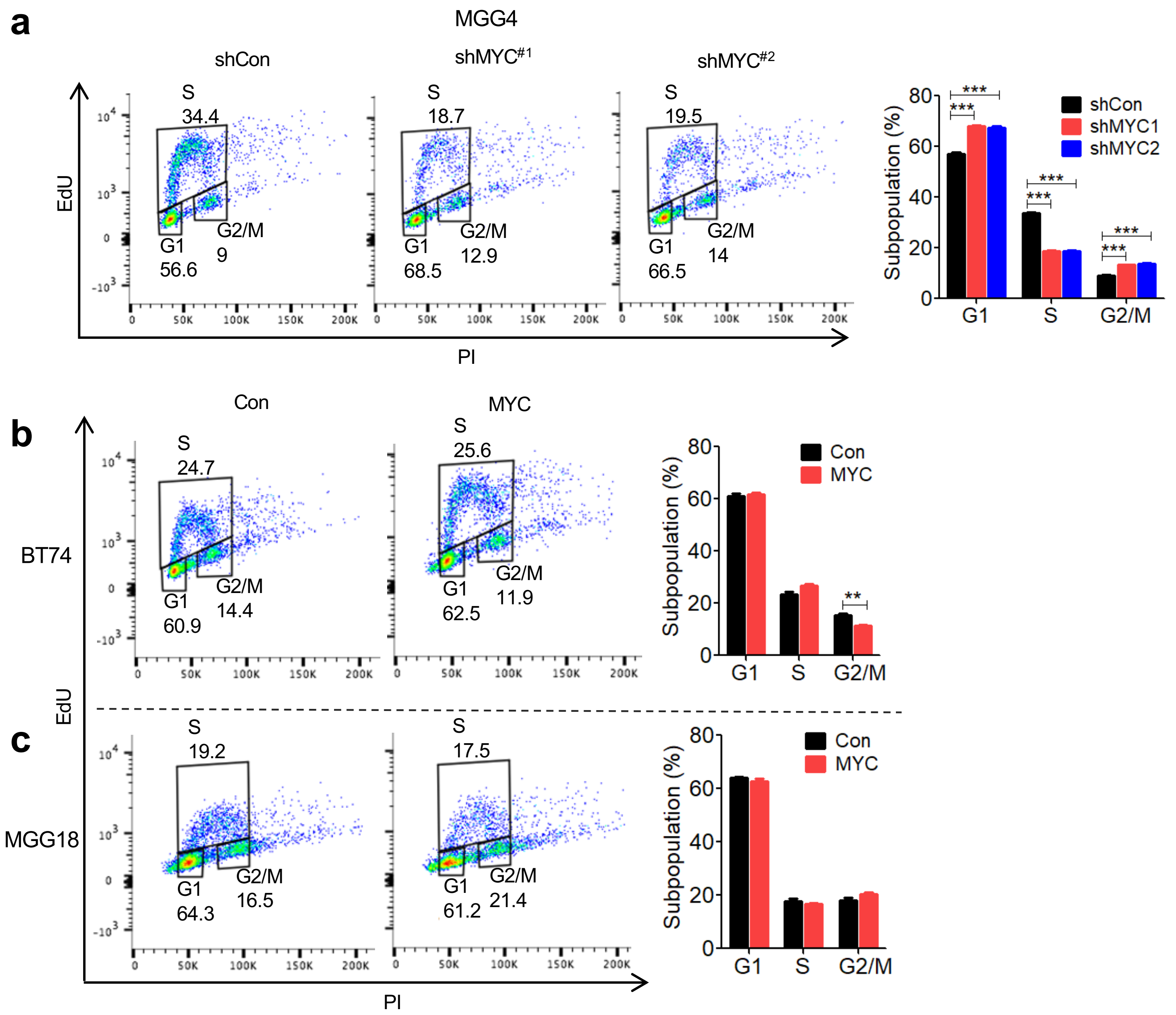

Supplementary Figure 2. Effect of MYC on cell cycle profiles in PARPi-sensitive and -resistant GSCs.

GSCs were pulse labeled with EdU (10 μM) for 30 min and stained with Propidium iodide (PI; 10 μg/ml) for cell cycle analysis by flow cytometry.

a. MGG4 with (shMYC; 2 independent sequences) or without (shCon) MYC knockdown.

b. BT74 with (MYC) or without (Con) MYC overexpression.

c. MGG18 with (MYC) or without (Con) MYC overexpression.

(Left) FACS plots. (Right) Quantification of cell cycle phases from Left. Data are mean ± SEM, n=3. **p<0.01, ***p<0.001, t-test.

Supplementary Figure 3

a

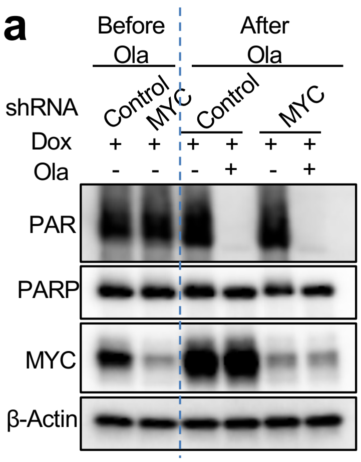

c

No.	Gene	shCon (Normalized)	shMYC (Normalized)	Folds (shMYC/shCon)	P values
1	CDK18	18.9561	157.4166	8.30	0
2	CCNE2	18.6396	49.1522	2.64	1.333E-168
3	CCNI2	2.098	5.2804	2.52	3.423E-16
4	CCPG1	1.8439	3.8598	2.09	2.163E-31
5	CDKN1A	2.1962	4.5457	2.07	1.892E-10
6	ATF3	1.5816	0.6681	0.42	5.561E-07
7	CCND2	10.1174	1.7646	0.17	1.505E-183

b

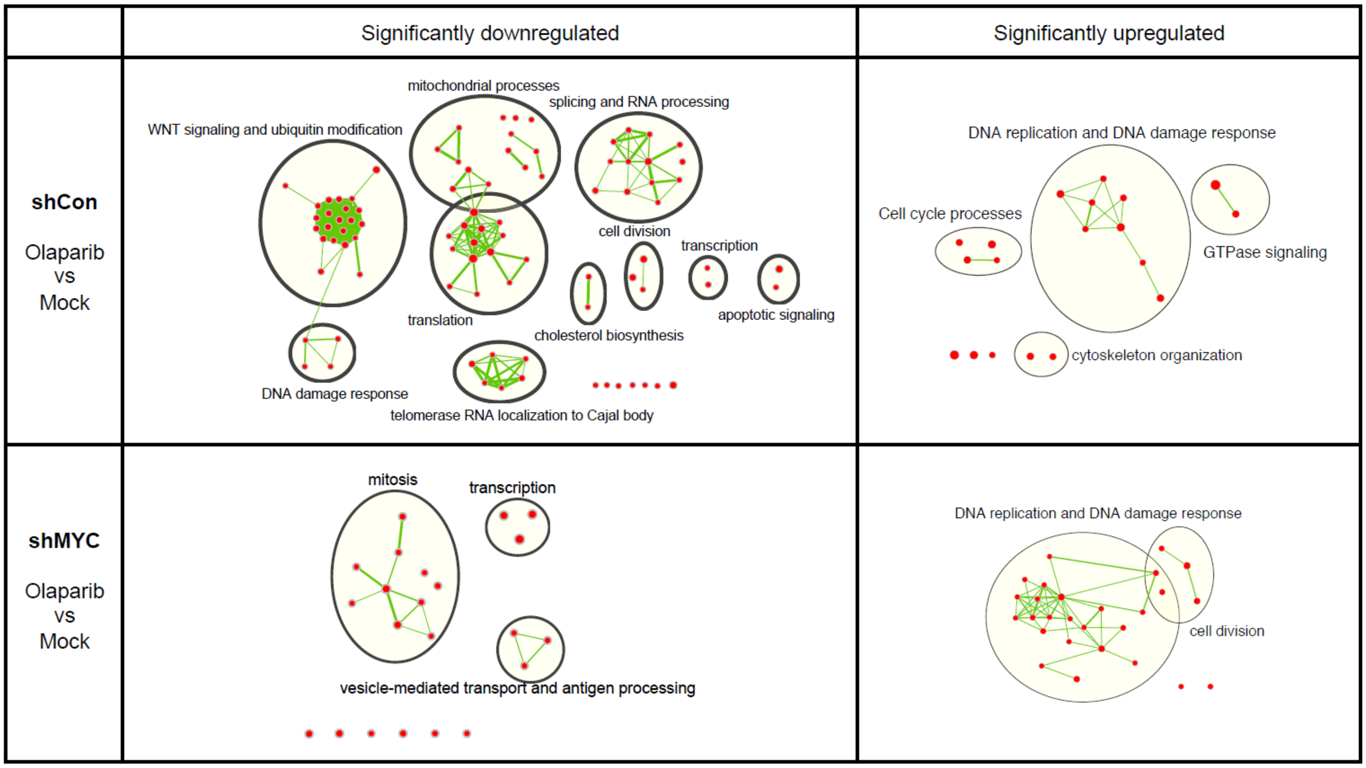

Supplementary Figure 3. RNA sequence profiling of transcripts altered by MYC knockdown in MGG4.

a. Western blot showing MYC knockdown and olaparib inhibition of PARP in MGG4 used for RNA-seq and quantitative RT-PCR. MGG4-shControl and -shMYC (shMYC#1) were grown in doxycycline (Dox, 1 μg/ml) for 6 days, treated with olaparib (Ola, 10 μM) for 24h, and harvested for western blot and RNA extraction. β-Actin was loading control.

b. Enrichment map of pathways altered by PARPi in MGG4-shMYC and -shControl (Con).

c. A list of DDR-associated genes whose transcript levels were changed by MYC knockdown over 2-fold with statistical significance ($p<0.05$, Chi-square test) and normalized value for shControl (Con) or shMYC >1.

Supplementary Figure 4

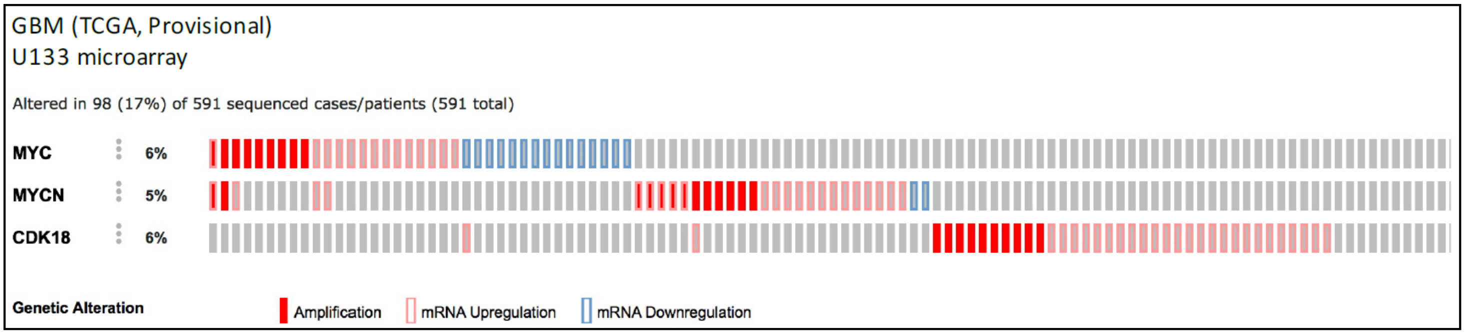

Supplementary Figure 4. Genetic alterations of MYC, MYCN and CDK18 in clinical GBM samples. Analysis of alterations of MYC, MYCN and CDK18 amplification and expression in GBM patient specimens from TCGA (Provisional) database through cBioPortal.

Supplementary Figure 5

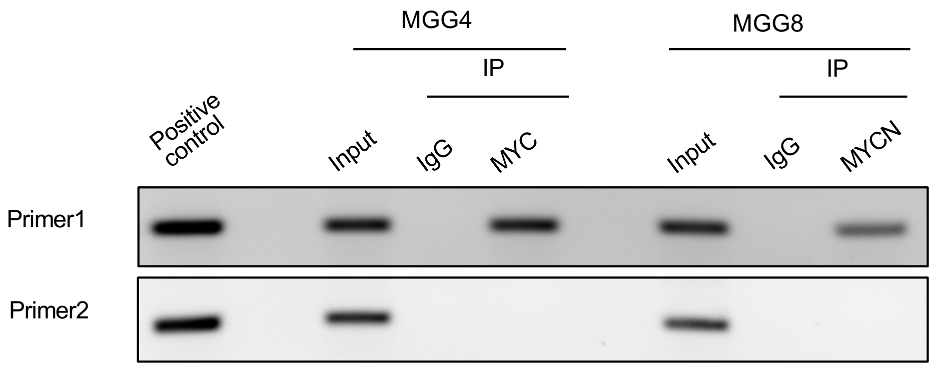

Supplementary Figure 5. Binding of Myc to its motif in CDK18 promoter. Products of chromatin immunoprecipitation (IP)-PCR from MGG4 and MGG8. Primer 1 and 2 from Fig. 2F. Positive control is MGG4 whole genomic DNA as template for PCR.

Supplementary Figure 6

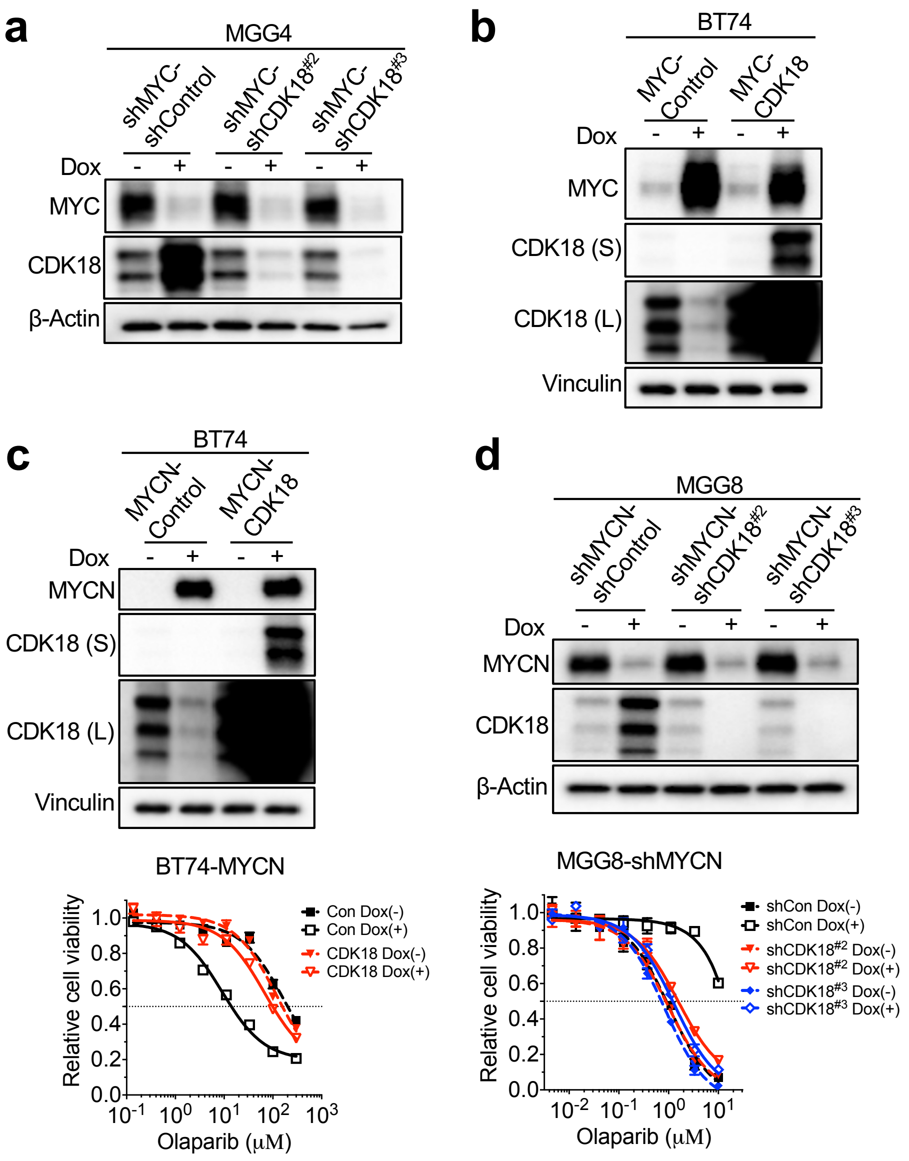

Supplementary Figure 6. CDK18 regulates MYC/MYCN-induced PARPi sensitivity in GSCs.

a. Representative western blot showing shRNA (sh) mediated knockdown of MYC alone and with CDK18 (2 separate shRNA sequences; same as Fig. 3) in MGG4 cells.

b. Representative western blot showing CDK18 and MYC expression in BT74 cells overexpressing MYC alone and with CDK18. Short (S) and long (L) exposure.

c. (Upper) Representative western blot showing CDK18 and MYCN expression in BT74 cells overexpressing MYCN alone and with CDK18. Short (S) and long (L) exposure. (Lower) Olaparib dose responses in BT74 overexpressing MYCN alone and with CDK18.

Supplementary Figure 6. ***Continued***

d. (Upper) Representative western blot showing shRNA (sh) knockdown of MYCN (#1) alone and with CDK18 (2 separate shRNA sequences; same as Fig. 3) in MGG8. (Lower) Olaparib dose responses in MGG8-shMYCN-shCDK18.

In **c** and **d**, MTS assay was performed at 6 days after olaparib treatment. Data normalized to relevant controls, mean ± SEM and representative of 3 independent experiments performed in triplicate. Dox(-)/(+): without/with doxycycline (1 μg/ml). Con: control. For western blots, cells were treated with (+) or without (-) Dox for 4 days. Vinculin or β-Actin are loading controls.

Supplementary Figure 7

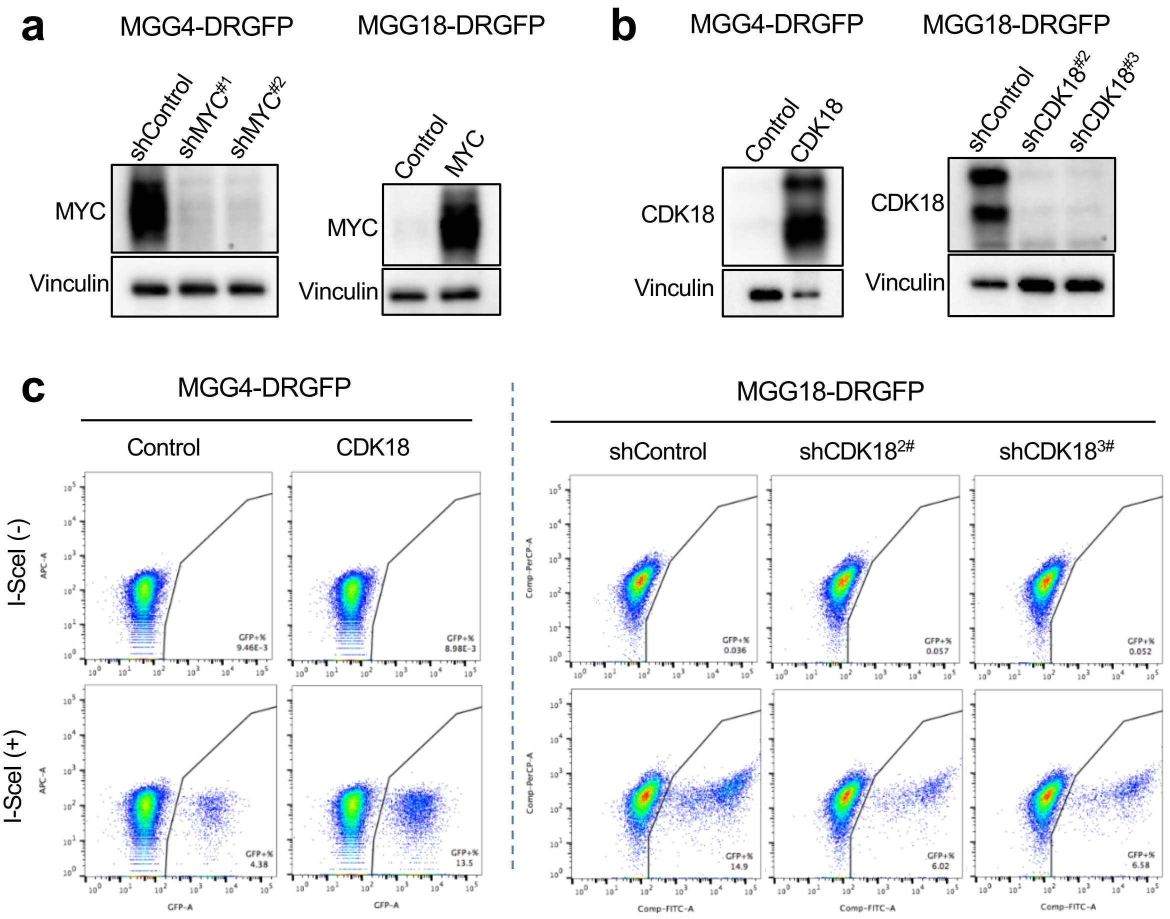

Supplementary Figure 7. MYC suppresses and CDK18 promotes homologous recombination (HR) in GSCs.

a. Representative western blots showing MYC knockdown (2 separate sequences; same as Fig. 1) in MGG4-DRGFP (left) or overexpression in MGG18-DRGFP (right).

b. Representative western blots showing CDK18 overexpression in MGG4-DRGFP (left) and knockdown (2 separate sequences) in MGG18-DRGFP (right).

c. Representative FACS plots for HR assay on MGG4-DRGFP with CDK18 overexpression (left) and on MGG18-DRGFP with CDK18 knockdown (right).

For western blot, cells were treated with doxycycline (1 μg/ml) for 4 days. For flow cytometry, cells were treated with doxycycline (1 μg/ml) for 6 days, followed by infection with lentivirus with (+) or without (-) I-SceI expression for 5 days, then sorted by flow cytometry to measure GFP$^+$ cells (right quadrant).

Supplementary Figure 8

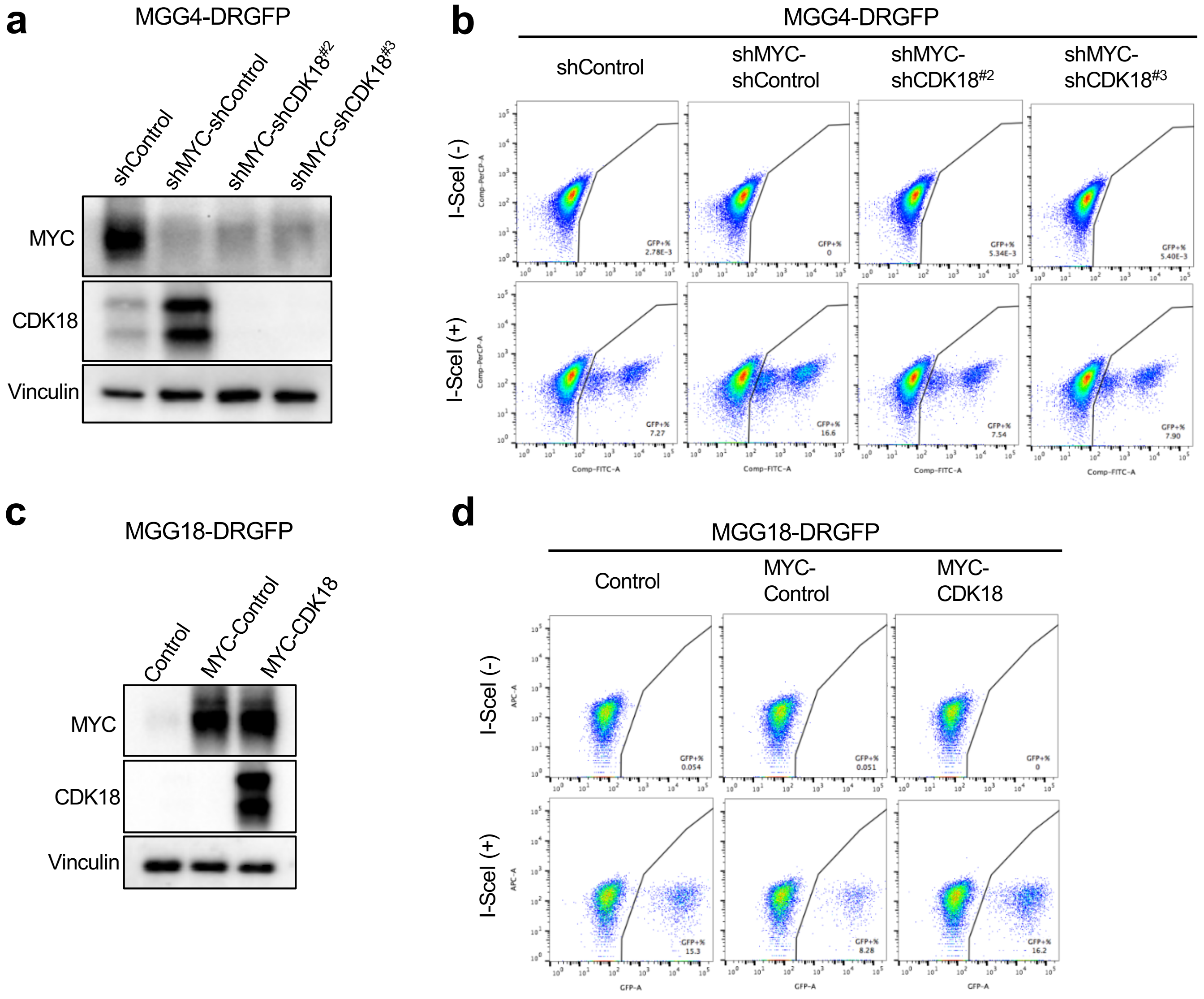

Supplementary Figure 8. MYC suppresses and CDK18 promotes homologous recombination (HR) in GSCs.

a. Representative western blot showing knockdown of MYC (shMYC#1) only and with CDK18 (2 separate sequences; same as Fig. 3) in MGG4-DRGFP.

b. Representative FACS plots for HR assay in MGG4-DRGFP with knockdown of MYC alone and with CDK18 (as in **a**).

c. Representative western blot showing MYC overexpression and with CDK18 in MGG18-DRGFP.

d. Representative FACS plots for HR assay in MGG18-DRGFP with overexpression of MYC alone and with CDK18 (as in **c**).

Cells were treated as in Supplementary Fig 7.

Supplementary Figure 9

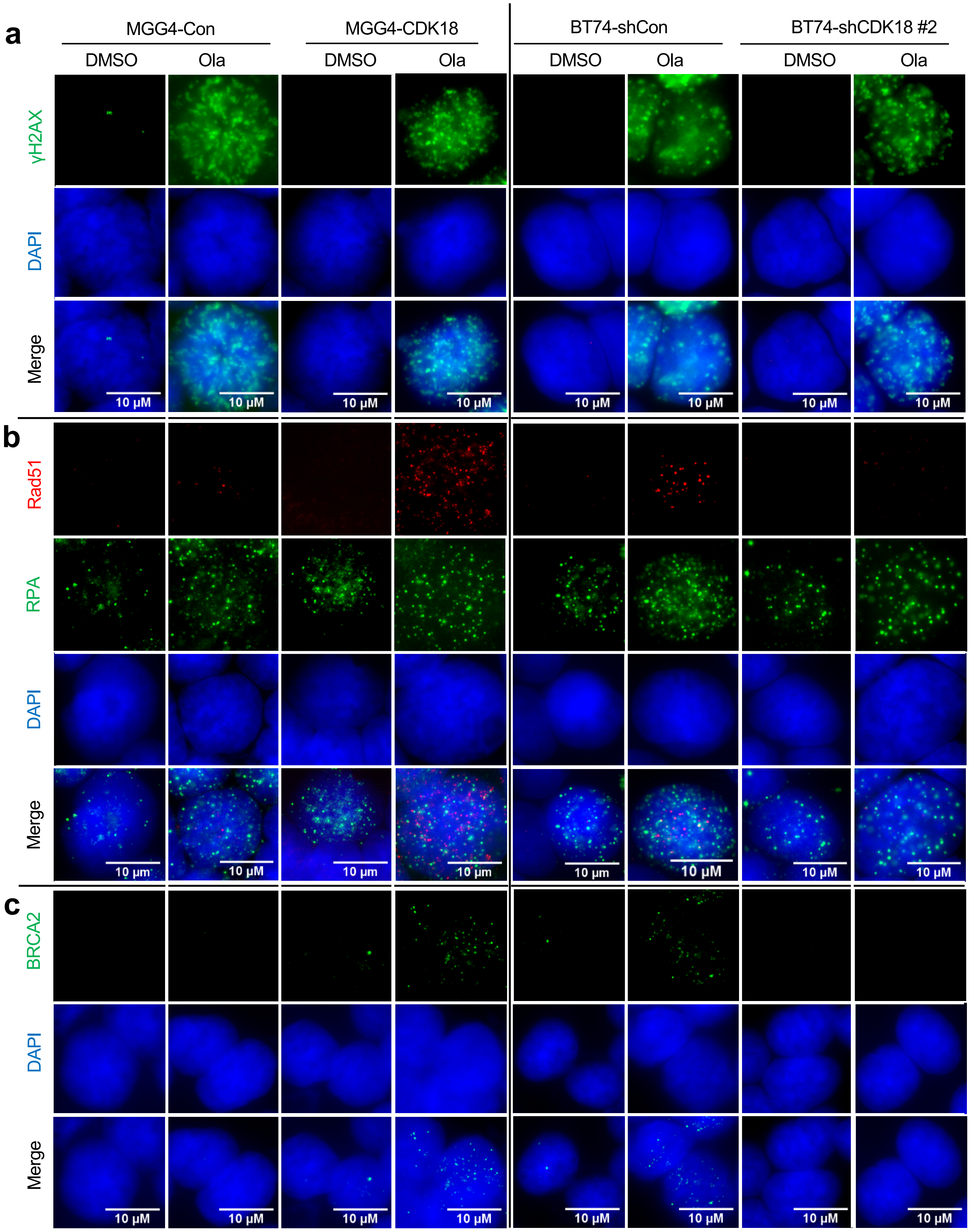

Supplementary Figure 9. CDK18 promotes homologous recombination (HR) repair in GSCs.

Representative immunofluorescence staining images showing γH2AX (**a**; green), RAD51/RPA (**b**; red/green), and BRCA2 (**c**; green) foci in GSCs with CDK18 overexpression (left) or knockdown (right). Cells were treated with olaparib (Ola, 15 μM) for 12 h before harvest for staining. Con, control. For quantification of the results, see Fig. 4e-g.

Supplementary Figure 10

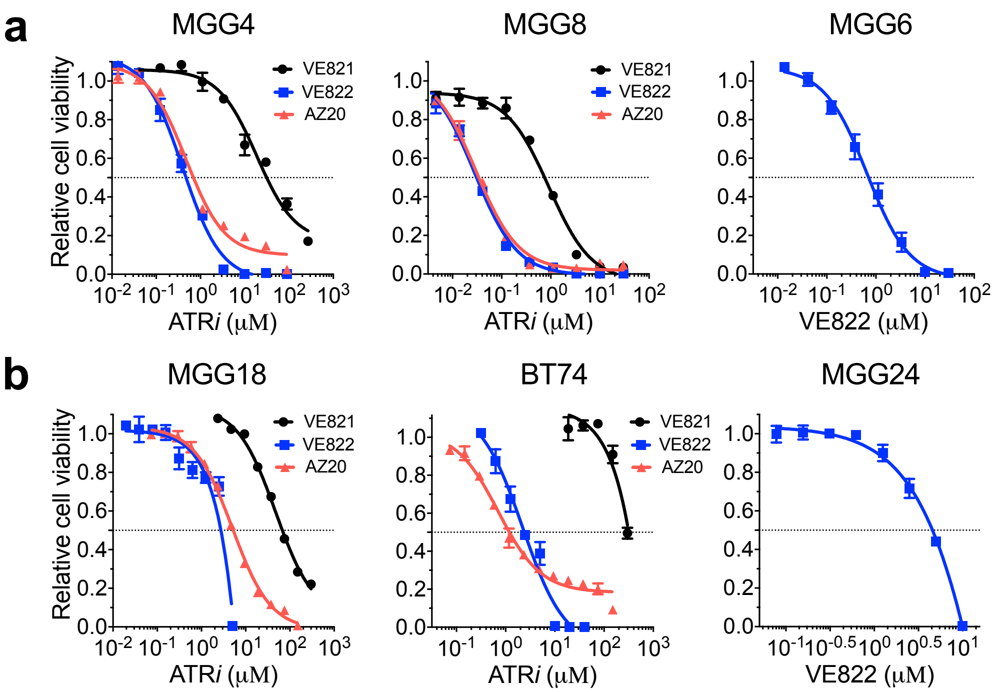

c

GSCs	IC50 (μM)		
	VE822	AZ20	VE821
MGG4	0.4±0.15	0.7±0.2	30±6
MGG8	0.03±0.01	0.04±0.02	0.9±0.25
MGG6	0.8±0.2	NT	NT
MGG18	3±1.4	5±1.9	65±12
BT74	2.2±1.6	1±0.6	300±103
MGG24	4±2	NT	NT

Supplementary Figure 10. Sensitivity of PARPi-sensitive and -resistant GSCs to ATR inhibitors.

a, b. Dose response curves for indicated ATR inhibitors (ATRis) on PARPi-sensitive GSCs (**a;** MGG4, MGG8 and MGG6) and -resistant GSCs (**b;** MGG18, BT74 and MGG24).

c. Half maximal inhibitory concentrations (IC50) for indicated ATRis in GSCs. Data were from dose response curves as shown in A and B. NT, not tested.

MTS assay was performed after 6-day treatment. Data were normalized to control and mean ± SEM, representative of three independent experiments performed in triplicate.

Supplementary Figure 11

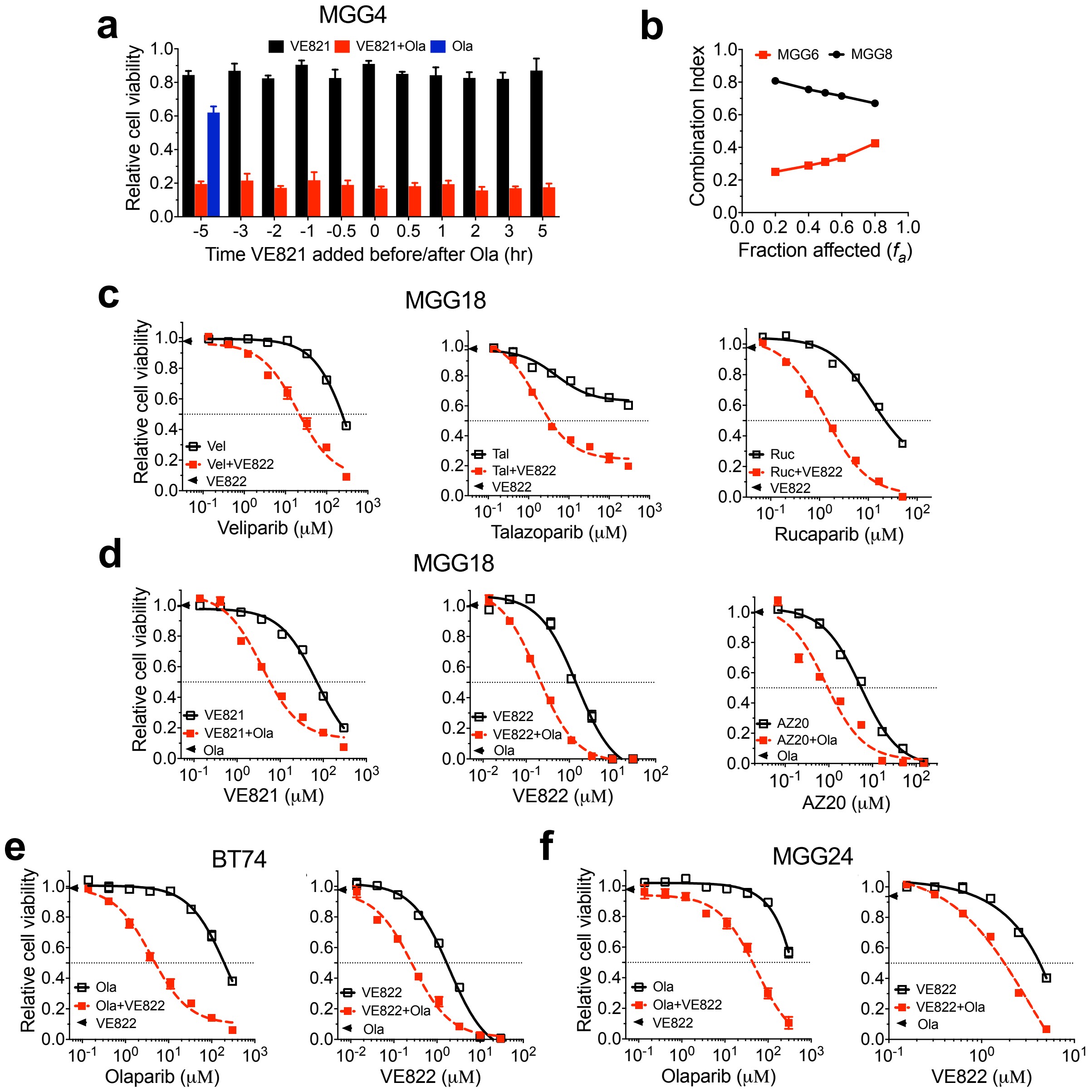

Supplementary Figure 11. ATR inhibitor synergizes with PARPi in killing PARPi-sensitive and -resistant GSCs.

a. Relative cell viability of MGG4 cells that were treated with ATRi VE821 (10 μM) at indicated times before (-) or after olaparib (Ola, 1 μM).

b. Chou-Talalay analysis showing the interaction between olaparib and VE822 in PARPi-sensitive MGG6 (red) and MGG8 (black). Combination Index <1 and >1 are synergistic and antagonistic, respectively.

c. Relative cell viability of MGG18 treated with different doses of indicated PARPis (Vel=veliparib, Tal=Talazoparib, Ruc=rucaparib) and with a fixed dose of VE822 (0.3 μM, red).

Supplementary Figure 11. ***Continued***

d. Relative cell viability of MGG18 treated with different doses of indicated ATRis and with a fixed dose of olaparib (Ola, 10 μM, red) .

e, f. Relative cell viability of BT74 (**e**) or MGG24 (**f**) treated with different doses of olaparib (Ola, left) and with a fixed dose of VE822 (0.5 μM, red), or different doses of VE822 (right) with a fixed dose of olaparib (10 μM, red).

In **c-f**, black triangle (◄) indicates viability with drug alone. MTS assay was performed after 6-day treatment. Data were normalized to control and mean ± SEM, representative of three independent experiments performed in triplicate.

Supplementary Figure 12

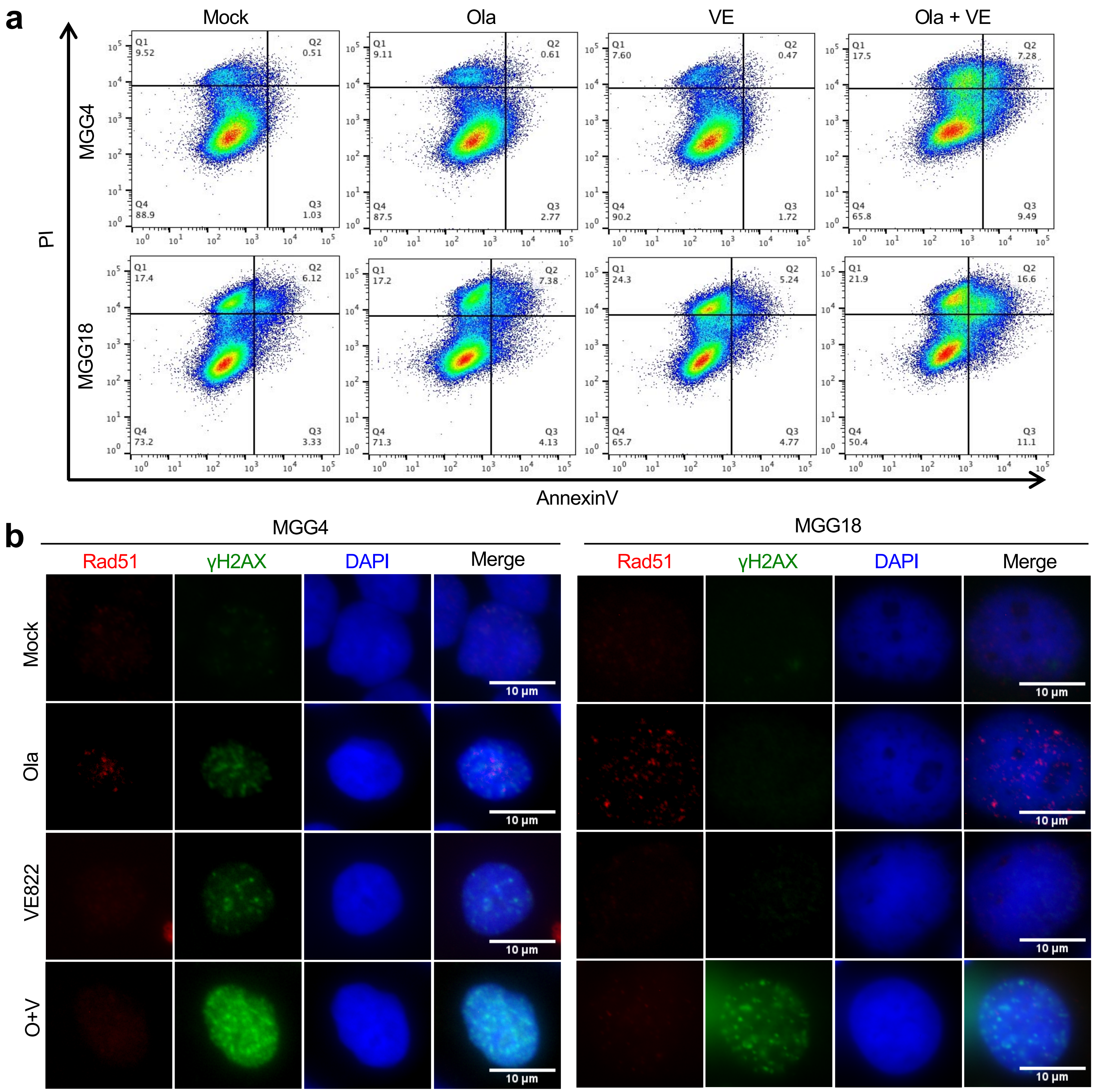

Supplementary Figure 12. ATRi inhibits Rad51 foci formation, and induces apoptosis and DNA damage in combination with PARPi.

a. Representative FACS plots showing apoptosis (right 2 quadrants) induced by ATRi and PARPi. MGG4 (upper panels) and MGG18 (lower panels) were treated for 72 h with olaparib (Ola; 3 and 10 μM) and/or VE822 (VE; 0.1 and 0.5 μM), respectively, and sorted for Annexin V / propidium iodide (PI) For quantification see Fig. 7f.

Supplementary Figure 12. *Continued*

b. Representative immunofluorescence staining images showing RAD51 (red) and γH2AX (green) foci in GSCs. (Left) MGG4 (PARPi-sensitive) treated with olaparib (Ola; 10 μM), VE822 (V; 0.3 μM), and combination (O+V). (Right) MGG18 (PARPi-resistant) treated with Ola (10 μM), VE822 (3 μM), and combination for 24 h before fixing and staining with antibodies and DAPI. For quantification see Fig. 7 g, h.

Supplementary Figure 13

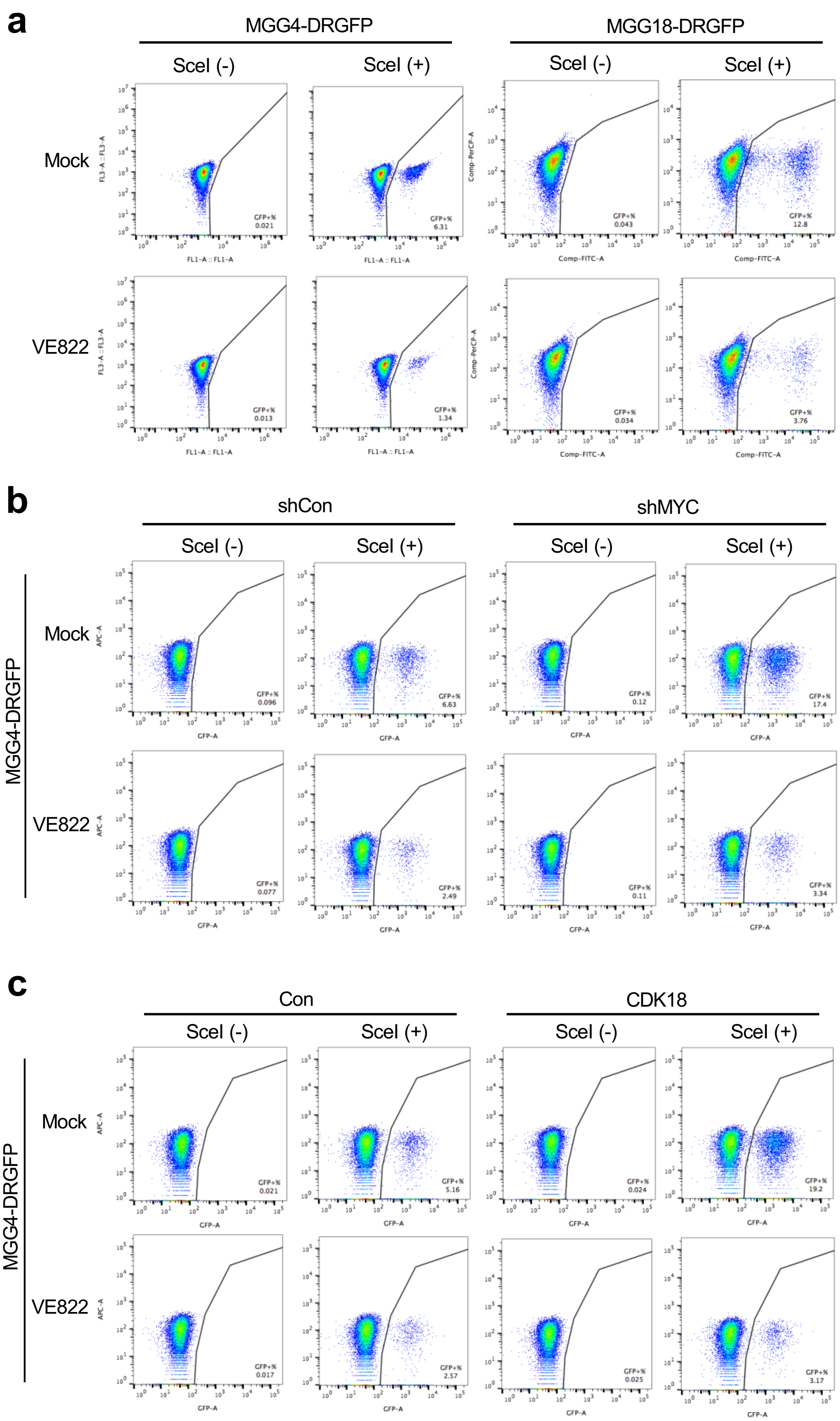

Supplementary Figure 13. Effects of ATRi on HR repair efficiency in GSCs with or without MYC knockdown or CDK18 overexpression.

a. Representative FACS plots for HR assay in MGG4- and MGG18-DRGFP cells infected with lentivirus expressing I-SceI (SceI (+)) or nothing (SceI (-)) for 4 days and then treated with DMSO (mock) or VE821 (0.1 μM) for 24h and sorted for GFP^+ cells (right quadrant).

b, c. Representative FACS plots for HR assay in MGG4-DRGFP with MYC knockdown (shMYC#1; **b**) or CDK18 overexpression **c**. Cells were treated with Dox (1 μg/ml) for 6 days and then subjected to procedures as in **a**.

Supplementary Figure 14

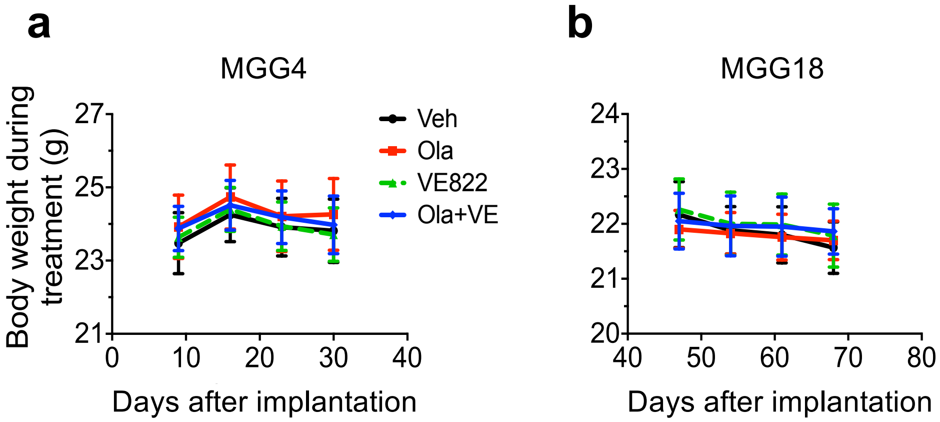

Supplementary Figure 14. ATRi is safely combined with PARPi in treating mice bearing GSC-derived tumors.

Body weight of mice bearing MGG4 (**a**) and MGG18 (**b**) tumors during treatment with olaparib (Ola), VE822, or combination (Ola+VE) from experiments in Fig. 8b-d. Data are mean ± SEM.

Supplementary Table 1

GSCs		Dox	IC50 (μM)	GSCs		Dox	IC50 (μM)
MGG4 (Fig. 1c)	parental	-	7.3	BT74 (Fig. 1e)	parental	-	130
	shCon	-	9		Con	-	135
		+	10			+	190
	shMYC$^{\#1}$	-	8		MYC	-	110
		+	110			+	14
	shMYC$^{\#2}$	-	8.9		MYCN	-	130
		+	80			+	12
MGG8 (Fig. 1d)	parental	-	0.85	MGG18 (Fig. S1h)	parental	-	310
	shCon	-	0.8		Con	-	300
		+	0.72			+	295
	shMYCN$^{\#1}$	-	0.61		MYC	-	305
		+	>10			+	30
	shMYCN$^{\#2}$	-	1		MYCN	-	290
		+	11			+	18

Supplementary Table 1. **Half maximal inhibitory concentrations (IC_{50}s) of olaparib for GSCs with different Myc status**. Representative IC_{50}s were derived from dose response curves in indicated figures.

Supplementary Table 2

GSCs		Dox	IC50 (μM)	GSCs		Dox	IC50 (μM)
MGG4 (Fig. 3a)	Con	-	9	MGG8 (Fig. 3a)	Con	-	0.9
		+	10.1			+	1
	CDK18	-	7		CDK18	-	1.1
		+	110			+	8
BT74 (Fig. 3b)	shCon	-	190	MGG18 (Fig. 3b)	shCon	-	320
		+	140			+	290
	shCDK18$^{\#2}$	-	170		shCDK18$^{\#2}$	-	300
		+	19			+	18
	shCDK18$^{\#3}$	-	180		shCDK18$^{\#3}$	-	335
		+	13			+	24
MGG4-shMYC (Fig. 3f)	shCon	-	8	MGG8-shMYCN (Fig. S6d)	shCon	-	0.9
		+	195			+	>10
	shCDK18$^{\#2}$	-	7.8		shCDK18$^{\#2}$	-	0.98
		+	8.9			+	1.3
	shCDK18$^{\#3}$	-	7		shCDK18$^{\#3}$	-	0.7
		+	10			+	1.1
BT74-MYC (Fig. 3g)	Con	-	185	BT74-MYCN (Fig. S6c)	Con	-	200
		+	9.5			+	13
	CDK18	-	184		CDK18	-	132
		+	226			+	107

Supplementary Table 2. Half maximal inhibitory concentrations (IC$_{50}$s) of olaparib for GSCs with knockdown or overexpression of Myc or/and CDK18. Representative IC$_{50}$s were derived from dose response curves in indicated figures.

Supplementary Table 3

Genes		shRNA sequences
MYC	1#	Forward 5’ CCGGCCTGAGACAGATCAGCAACAACTCGAGTTGTTGCTGATCTGT CTCAGGTTTTT 3’
		Reverse 5’ AATTAAAAACCTGAGACAGATCAGCAACAACTCGAGTTGTTGCTGAT CTGTCTCAGG 3’
	2#	Forward 5’ CCGGGCTTCACCAACAGGAACTATGCTCGAGCATAGTTCCTGTTGG TGAAGCTTTTT 3’
		Reverse 5’ AATTAAAAAGCTTCACCAACAGGAACTATGCTCGAGCATAGTTCCTG TTGGTGAAGC 3’
MYCN	1#	Forward 5’ CCGGCAGCAGCAGTTGCTAAAGAAACTCGAGTTTCTTTAGCAACTG CTGCTGTTTTT 3’
		Reverse 5’ AATTAAAAACAGCAGCAGTTGCTAAAGAAACTCGAGTTTCTTTAGCA ACTGCTGCTG 3’
	2#	Forward 5’ CCGGCTGAGCGATTCAGATGATGAACTCGAGTTCATCATCTGAATC GCTCAGTTTTT 3’
		Reverse 5’ AATTAAAAACTGAGCGATTCAGATGATGAACTCGAGTTCATCATCTG AATCGCTCAG 3’
CDK18	2#	Forward 5’ CCGGGAACCTGAAGCACGCCAATATCTCGAGATATTGGCGTGCTTC AGGTTCTTTTT 3’
		Reverse 5’ AATTAAAAAGAACCTGAAGCACGCCAATATCTCGAGATATTGGCGTG CTTCAGGTTC 3’
	3#	Forward 5’ CCGGGCATGCACAACGTCAAGATTTCTCGAGAAATCTTGACGTTGT GCATGCTTTTT
		Reverse 5’ AATTAAAAAGCATGCACAACGTCAAGATTTCTCGAGAAATCTTGAC GTTGTGCATGC 3’
scrambled		Forward 5’ CCGGGCTTCGCGCCGTAGTCTTACTCGAGTAAGACTACGGCGCGA AGCTTTTT 3’
		Reverse AATTAAAAAGCTTCGCGCCGTAGTCTTACTCGAGTAAGACTACGGCG CGAAGC 3’

Supplementary Table 3. **DNA oligonucleotides used for shRNA studies**. Oligonucleotides were synthesized by Thermo Fisher Scientific.

Supplementary Table 4

cDNA	Primers
MYC	Forward 5’ CTTACTAGTCACCATGCCCCTCAACGTTAGCTT 3’
	Reverse 5’ CTTTTCGAATTACGCACAAGAGTTCCGTAG 3’
MYCN	Forward 5’ GCGACTAGTCACCATGCCGAGCTGCTCCACGTCCACCATG 3’
	Reverse 5’ GGTAATTCGAACTAGCAAGTCCGAGCGTGTTCAATTTTCT 3’
CDK18	Forward 5’ GGGGACAAGTTTGTACAAAAAAGCAGGCTTCACCATGAACAAGATGAAGA ACTTTAAGC 3’
	Reverse 5’ GGGGACCACTTTGTACAAGAAAGCTGGGTCCTATCAGAAGATGCTCTGCC GCCTGTTC 3’

Supplementary Table 4. **DNA oligonucleotides used to clone cDNAs**. Oligonucleotides were synthesized by Thermo Fisher Scientific.

Supplementary Table 5

Target of antibody	Dilution	Type	Source
PAR	1:10,000	Rabbit polyclonal	Trevigen
PARP	1:3000	Rabbit polyclonal	CST
Cleaved-PARP	1:3000	Rabbit polyclonal	CST
Cleaved-caspase 3	1:1000	Rabbit polyclonal	CST
γ-H2AX	1:1000	Rabbit monoclonal	CST
Phospho-ATM (Ser1981)	1:10,000	Mouse monoclonal	CST
ATM	1:10,000	Rabbit monoclonal	CST
Phospho-ATR (Ser428)	1:1000	Rabbit polyclonal	CST
ATR	1:1000	Rabbit polyclonal	Bethyl
p-ATR (T1889)	1:1000	Rabbit polyclonal	GeneTex
TopBP1	1:1000	Rabbit polyclonal	Bethyl
Rad9	1:1000	Rabbit polyclonal	Bethyl
ETAA1	1:1000	Rabbit polyclonal	Abcam
Phospho-Chk1 (Ser345)	1:1000	Rabbit monoclonal	CST
Chk1	1:1000	Mouse monoclonal	Santa Cruz
Rad51	1:1000	Rabbit monoclonal	CST
Rad17	1:1000	Rabbit polyclonal	Bethyl
Phospho-Rad17	1:1000	Rabbit monoclonal	CST
BRCA1	1:1000	Rabbit polyclonal	CST
BRCA2	1:1000	Mouse monoclonal	Millipore
CDK18	1:1000	Rabbit polyclonal	Santa Cruz
CDK18	1:1000	Mouse monoclonal	Santa Cruz
MYC	1:1000	Rabbit polyclonal	CST
MYCN	1:1000	Rabbit polyclonal	CST
PTEN	1:1000	Rabbit polyclonal	CST
EGFR	1:1000	Rabbit polyclonal	CST
Phospho-EGFR	1:1000	Rabbit polyclonal	CST
Akt	1:1000	Rabbit polyclonal	CST
Phospho-Akt	1:1000	Rabbit polyclonal	CST
GAPDH	1:10,000	Mouse monoclonal	Acris
Vinculin	1:10,000	Mouse monoclonal	Thermo
β-Actin	1:10,000	Rabbit polyclonal	Sigma

Supplementary Table 5. Primary antibodies used in this study. Antibody Sources: Acris, Acris Antibodies, San Diego CA; Bethyl, Bethyl Laboratories, Montgomery TX; CST, Cell Signaling Technology, Danvers MA; Millipore, MilliporeSigma, Bedford, MA; Santa Cruz, Santa Cruz Biotechnology, Dallas TX; Sigma, St Louis MO; Thermo, Thermo Scientific, Rockford IL; Trevigen, Gaithersburg MD.
