# Supplementary material for: Myc targeted CDK18 promotes ATR and homologous recombination to mediate PARP inhibitor resistance in glioblastoma
Source: Nat Commun. 2019 Jul 2;10:2910. doi: 10.1038/s41467-019-10993-5 (PMC6606647; doi:10.1038/s41467-019-10993-5)
Supplement: Supplementary file 2 — Reporting Summary [file 41467_2019_10993_MOESM2_ESM.pdf]

nature research

Corresponding author(s): Samuel D. Rabkin
Last updated by author(s): May 31, 2019

Reporting Summary

Nature Research wishes to improve the reproducibility of the work that we publish. This form provides structure for consistency and transparency in reporting. For further information on Nature Research policies, see Authors & Referees and the Editorial Policy Checklist.

Statistics

For all statistical analyses, confirm that the following items are present in the figure legend, table legend, main text, or Methods section.

n/a	Confirmed	
☐	☒	The exact sample size (*n*) for each experimental group/condition, given as a discrete number and unit of measurement
☐	☒	A statement on whether measurements were taken from distinct samples or whether the same sample was measured repeatedly
☐	☒	The statistical test(s) used AND whether they are one- or two-sided<br>*Only common tests should be described solely by name; describe more complex techniques in the Methods section.*
☐	☒	A description of all covariates tested
☐	☒	A description of any assumptions or corrections, such as tests of normality and adjustment for multiple comparisons
☐	☒	A full description of the statistical parameters including central tendency (e.g. means) or other basic estimates (e.g. regression coefficient) AND variation (e.g. standard deviation) or associated estimates of uncertainty (e.g. confidence intervals)
☐	☒	For null hypothesis testing, the test statistic (e.g. *F*, *t*, *r*) with confidence intervals, effect sizes, degrees of freedom and *P* value noted<br>*Give P values as exact values whenever suitable.*
☐	☒	For Bayesian analysis, information on the choice of priors and Markov chain Monte Carlo settings
☐	☒	For hierarchical and complex designs, identification of the appropriate level for tests and full reporting of outcomes
☐	☒	Estimates of effect sizes (e.g. Cohen's *d*, Pearson's *r*), indicating how they were calculated

Our web collection on statistics for biologists contains articles on many of the points above.

Software and code

Policy information about availability of computer code

Data collection	No software was used
Data analysis	PRISM 5.0, ImageJ (Fiji) and FlowJo (X)

For manuscripts utilizing custom algorithms or software that are central to the research but not yet described in published literature, software must be made available to editors/reviewers. We strongly encourage code deposition in a community repository (e.g. GitHub). See the Nature Research guidelines for submitting code & software for further information.

Data

Policy information about availability of data

All manuscripts must include a data availability statement. This statement should provide the following information, where applicable:

- Accession codes, unique identifiers, or web links for publicly available datasets
- A list of figures that have associated raw data
- A description of any restrictions on data availability

Patient-derived glioblastoma stem cells are available to research community after MTA.

Field-specific reporting

Please select the one below that is the best fit for your research. If you are not sure, read the appropriate sections before making your selection.

☒ Life sciences ☐ Behavioural & social sciences ☐ Ecological, evolutionary & environmental sciences

For a reference copy of the document with all sections, see nature.com/documents/nr-reporting-summary-flat.pdf

Life sciences study design

All studies must disclose on these points even when the disclosure is negative.

Sample size	N=8 mice/group in in vivo survival studies was determined based on power analysis on SD of our previous experiments using the same models.
Data exclusions	No data was excluded from the study.
Replication	Results were repeated 2-3 times
Randomization	In all in vivo studies, mice with established brain tumors were randomized to groups that are assigned to different treatments
Blinding	Animal facility staff that monitored symptoms were blinded to treatments.

Reporting for specific materials, systems and methods

We require information from authors about some types of materials, experimental systems and methods used in many studies. Here, indicate whether each material, system or method listed is relevant to your study. If you are not sure if a list item applies to your research, read the appropriate section before selecting a response.

Materials & experimental systems

n/a	Involved in the study	
☐	☒	Antibodies
☐	☒	Eukaryotic cell lines
☒	☐	Palaeontology
☐	☒	Animals and other organisms
☒	☐	Human research participants
☒	☐	Clinical data

Methods

n/a	Involved in the study	
☒	☐	ChIP-seq
☐	☒	Flow cytometry
☒	☐	MRI-based neuroimaging

Antibodies

Antibodies used	PARP, Cleaved-PARP (Asp214), Cleaved Caspase-3 (Asp175), Phospho-Histone H2A.X (Ser139) (20E3), Phospho-Chk1 (Ser345) (133D3), Rad51 (D4B10), BRCA1, c-Myc, N-Myc, Chk1 (G-4), PCTAIRE-3 (C-17), RPA, PAR, ATR, BRCA2 (Ab-1), GAPDH, Vinculin, β-Actin, p-ATR (T1889), TopBP1, Rad9, ETAA1, Rad17, Phospho-Rad17
Validation	PARP (1:3000, Rabbit polyclonal, #9542), Cleaved-PARP (Asp214) (human, 1:3000, Rabbit polyclonal, #9541), Cleaved Caspase-3 (Asp175) (1:1000, Rabbit polyclonal, #9661), Phospho-Histone H2A.X (Ser139) (20E3) (1:1000, Rabbit monoclonal, #9718), Phospho-Chk1 (Ser345) (133D3) (1:1000, Rabbit monoclonal, #2348), Rad51 (D4B10) (1:1000, Rabbit monoclonal, #8875), BRCA1 (1:1000, Rabbit polyclonal, #9010), c-Myc (1:1000, Rabbit polyclonal, #9402), N-Myc (1:1000, Rabbit polyclonal, #9405) and Phospho-Rad17 (Ser645) (1:1000, Rabbit monoclonal, #6981) were from Cell Signaling Technology (Danvers MA).<br>Chk1 (G-4) (1:1000, Mouse monoclonal, sc-8408) and PCTAIRE-3 (C-17) (1:1000, Rabbit polyclonal, # sc-176), PCTAIRE-3 (H-4) (1:1000, mouse monoclonal , #sc-393262),RPA 32 kDa subunit Antibody (9H8) (1:200, mouse monoclonal, sc-56770) were from Santa Cruz Biotechnology (Dallas TX).<br>ATR(1:1000, Rabbit polyclonal, # A300-138A), Rad17(1:1000, Rabbit polyclonal, A305-788A-M), TopBP1 (1:1000, Rabbit polyclonal, A300-111A), Rad9 (1:1000, Rabbit polyclonal, A300-890A) were from Bethyl Laboratories (Montgomery TX)<br>PAR (1:10,000, Rabbit polyclonal, # 4336-BPC-100) from Trevigen (Gaithersburg MD), BRCA2 (Ab-1) (1:1000, Mouse monoclonal, # OP95) from Millipore, GAPDH (1:10,000, Mouse monoclonal, clone OTI2D9 , # TA802519) from Acris Antibodies (San Diego CA), Vinculin (1:10,000, Mouse Monoclonal, # MA5-11690) from Thermo Scientific (Rockford IL), β-Actin (1:10,000, Rabbit polyclonal, # A2066) from Sigma (Bedford, MA), p-ATR (T1889)(1:1000, Rabbit polyclonal, GTX128145) from GeneTex and ETAA1 (1:1000, Rabbit polyclonal, ab192402) from Abcam.

Eukaryotic cell lines

Policy information about cell lines

Cell line source(s)	Human GSCs MGG4, MGG6, MGG8, MGG152, MGG13, MGG18, MGG24 were isolated from GBM patient specimens in the lab and BT74 was from Dr. Santosh Kesari. 293T cells was obtained from American Type Culture Collection (ATCC, Manassas, VA), and normal human astrocytes from ScienCell (Carlsbad, CA).
Authentication	Genetic analysis confirmed the presence of patient-specific driver mutations or gene amplification in human GSCs. 293T and NHA have not been authenticated.
Mycoplasma contamination	The cell lines tested negative for mycoplasma.

Commonly misidentified lines (See ICLAC register)	No

Animals and other organisms

Policy information about studies involving animals; ARRIVE guidelines recommended for reporting animal research

Laboratory animals	SCID and athymic mice, 7-8 wekks, female
Wild animals	The study did not involve wild animals
Field-collected samples	The study did not involve samples collected from field
Ethics oversight	All in vivo procedures were approved by the Institutional Animal Care and Use Committee at Massachusetts General Hospital.

Note that full information on the approval of the study protocol must also be provided in the manuscript.

Flow Cytometry

Plots

Confirm that:

☒ The axis labels state the marker and fluorochrome used (e.g. CD4-FITC).

☒ The axis scales are clearly visible. Include numbers along axes only for bottom left plot of group (a 'group' is an analysis of identical markers).

☒ All plots are contour plots with outliers or pseudocolor plots.

☒ A numerical value for number of cells or percentage (with statistics) is provided.

Methodology

Sample preparation	Human glioblastoma cells in culture were spun down and manually processed for staining or fixation
Instrument	Sorvall ST 40 Centrifuge
Software	FlowJo (X)
Cell population abundance	Cells were gated with purity over 85% by excluding debris
Gating strategy	Single cells were selected by FSC/SSC, FSC-H/FSC-A and SSC-H/FSC-A gates. Boundaries between “negative” and “positive” staining cell populations were determined by negative and positive staining control.

☒ Tick this box to confirm that a figure exemplifying the gating strategy is provided in the Supplementary Information.
