# Supplementary material for: Myc targeted CDK18 promotes ATR and homologous recombination to mediate PARP inhibitor resistance in glioblastoma
Source: Nat Commun. 2019 Jul 2;10:2910. doi: 10.1038/s41467-019-10993-5 (PMC6606647; doi:10.1038/s41467-019-10993-5)
Supplement: Supplementary file 3 — Source Data [file 41467_2019_10993_MOESM3_ESM.pdf]

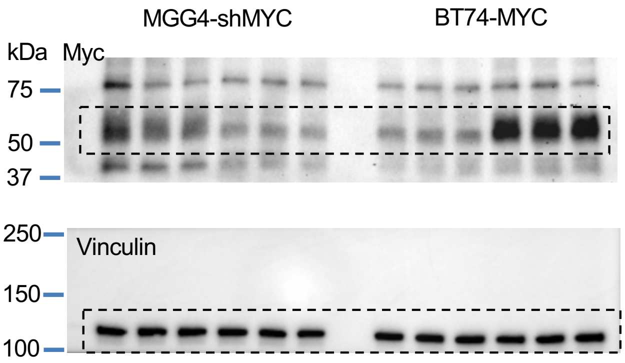

Source data 1. Uncropped western blots for Fig 1f. Dotted lines indicate cropped area.

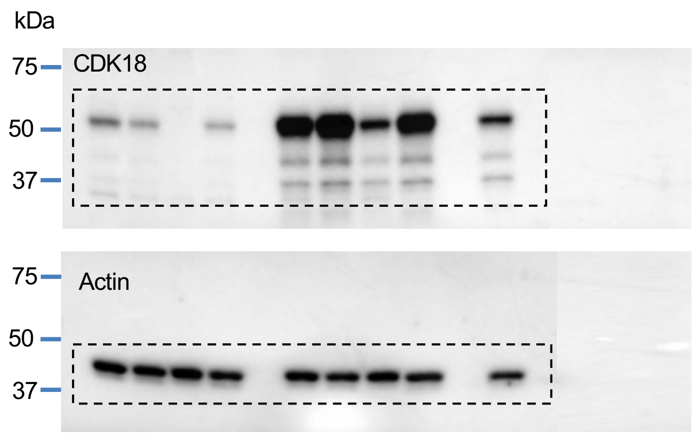

Source data 2. Uncropped western blots for Fig 2c. Dotted lines indicate cropped area. Unrelated blots on the right are covered.

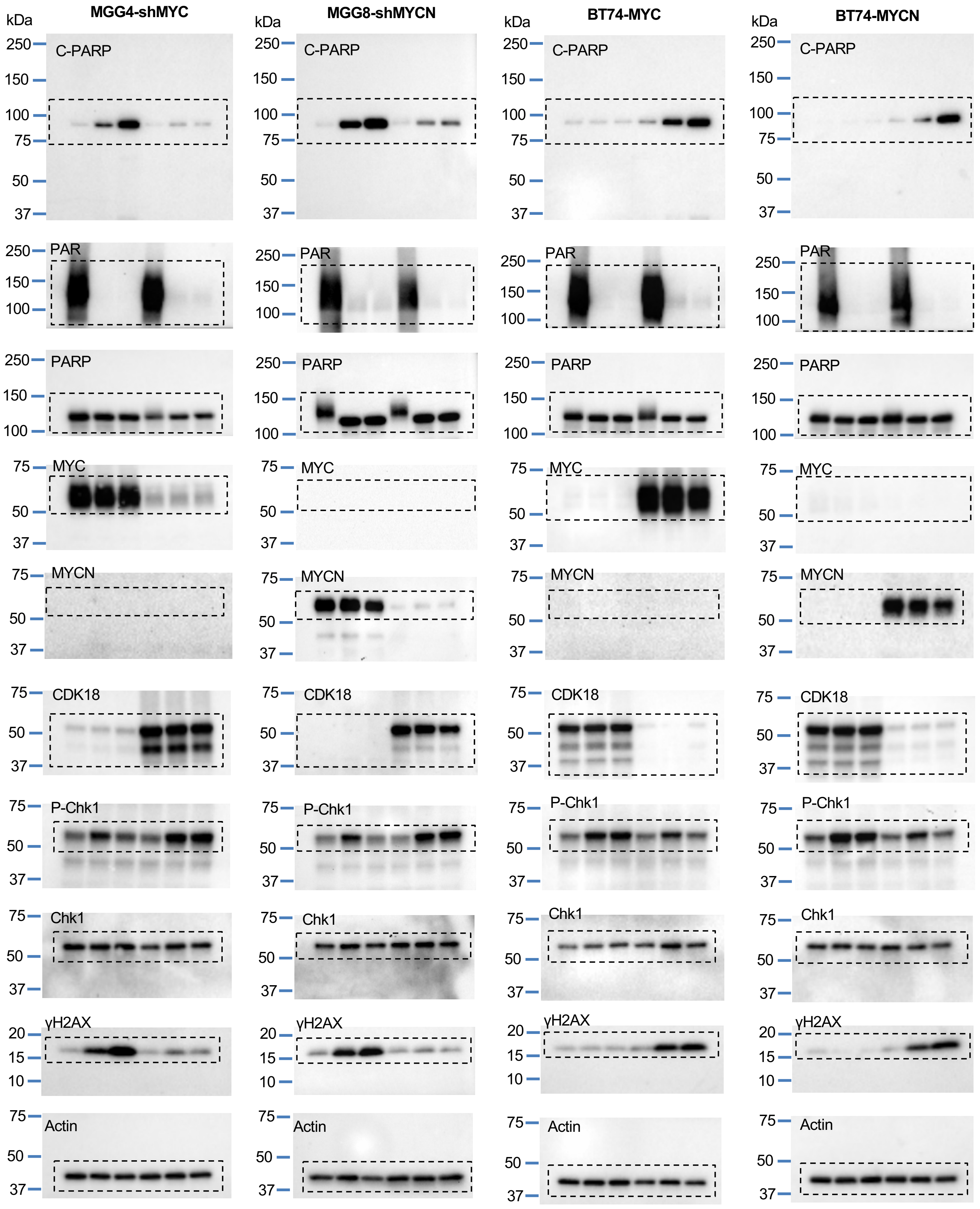

Source data 3. Uncropped western blots for Fig 2d and Fig 5a. Dotted lines indicate cropped area.

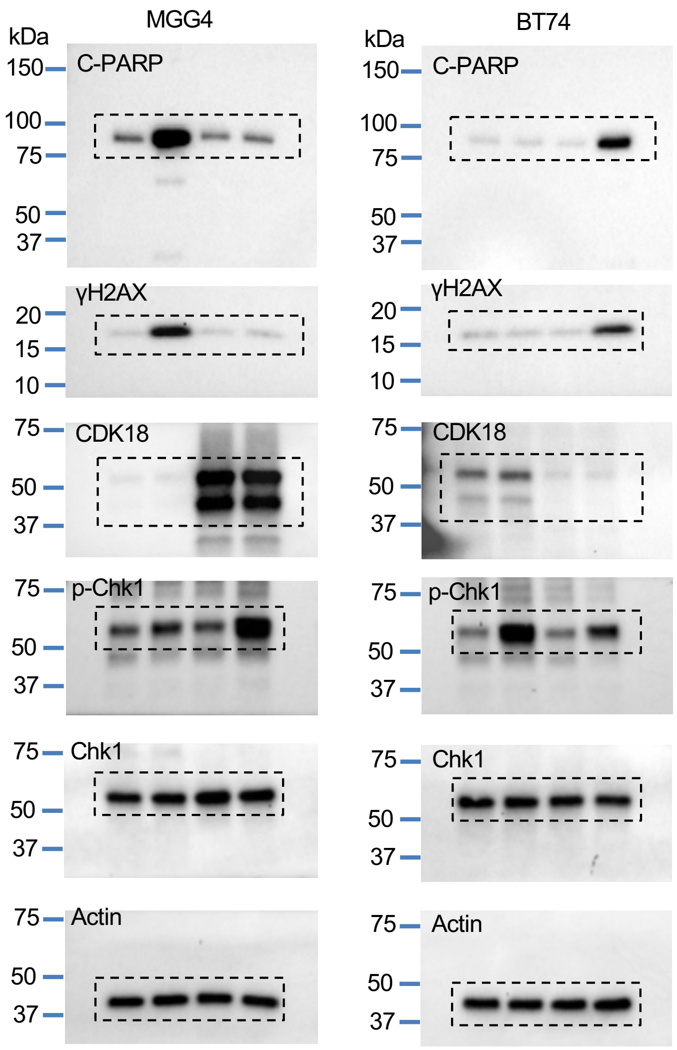

Source data 4. Uncropped western blots for Fig 5d. Dotted lines indicate cropped area.

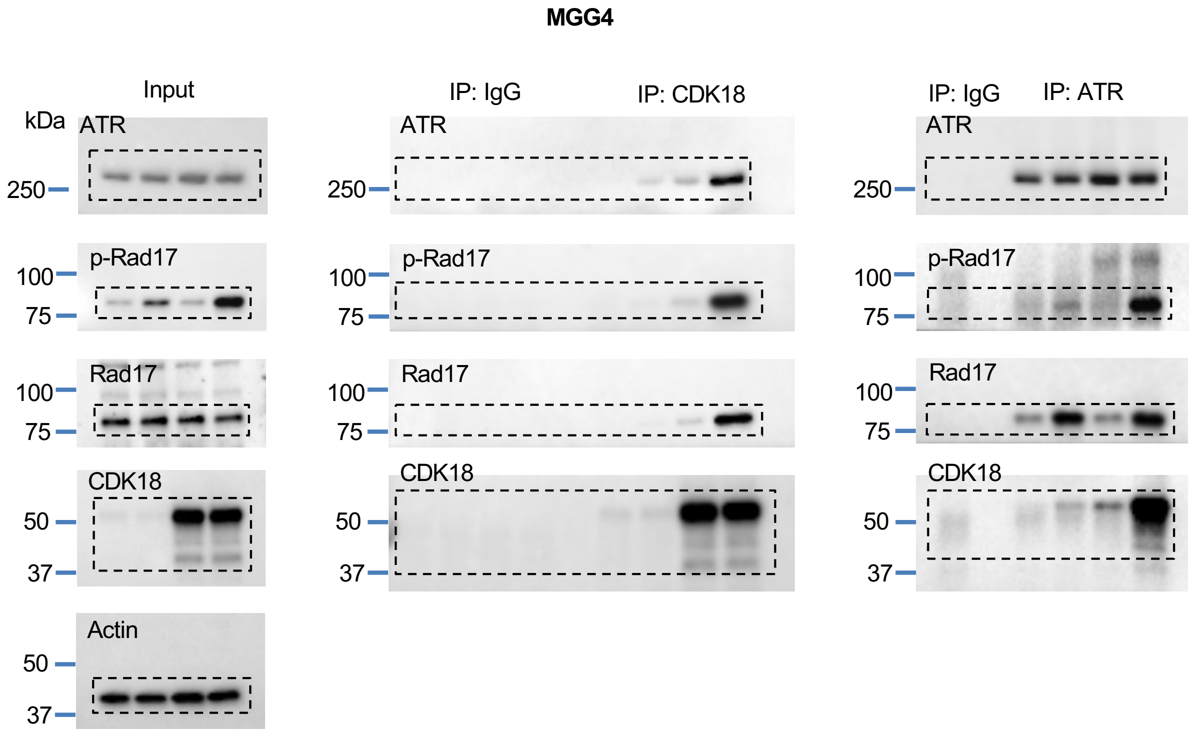

Source data 5. Uncropped western blots for Fig 6a . Dotted lines indicate cropped area.

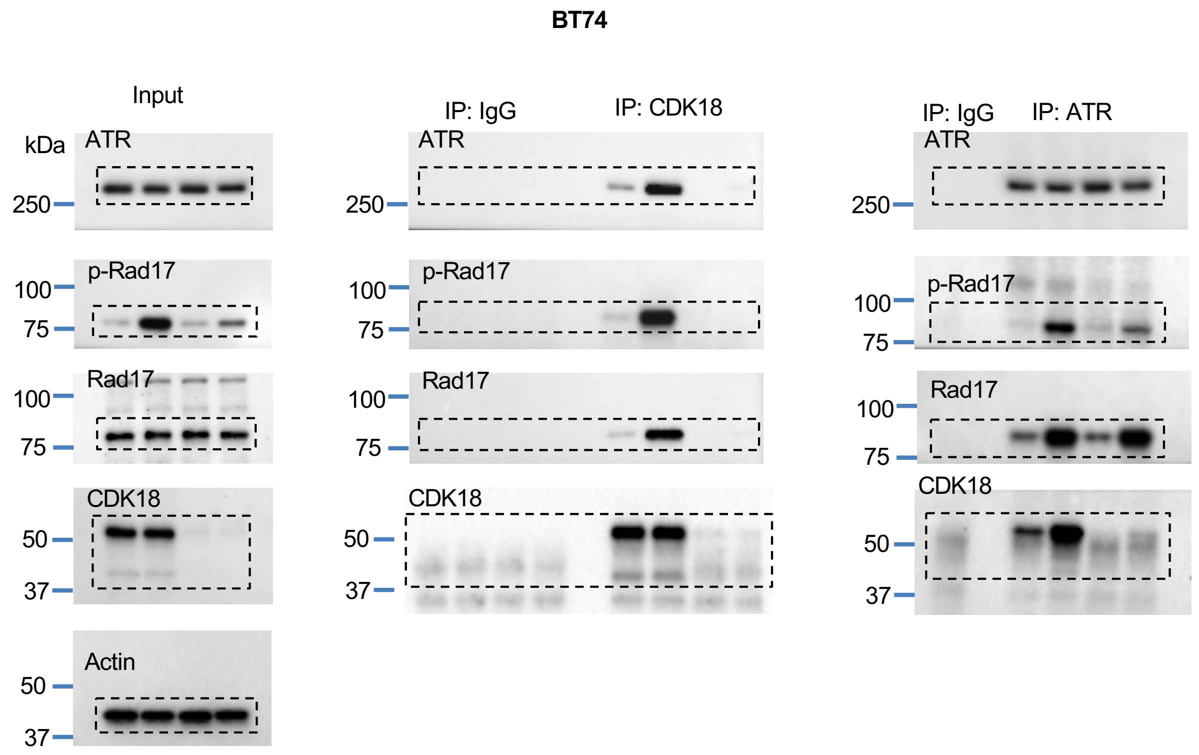

Source data 6. Uncropped western blots for Fig6b. Dotted lines indicate cropped area.

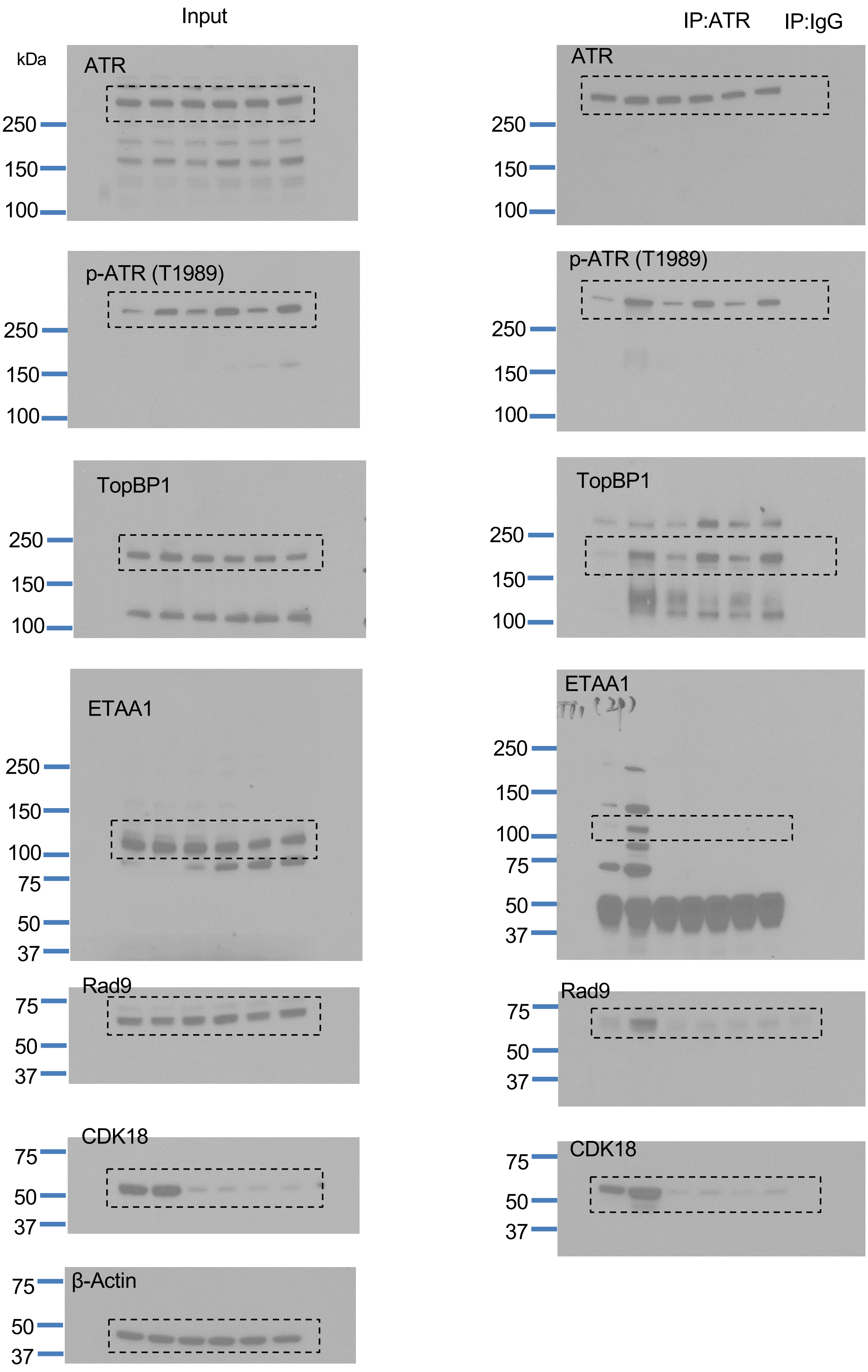

Source data 7. Uncropped western blots for Fig6c. Dotted lines indicate cropped area.

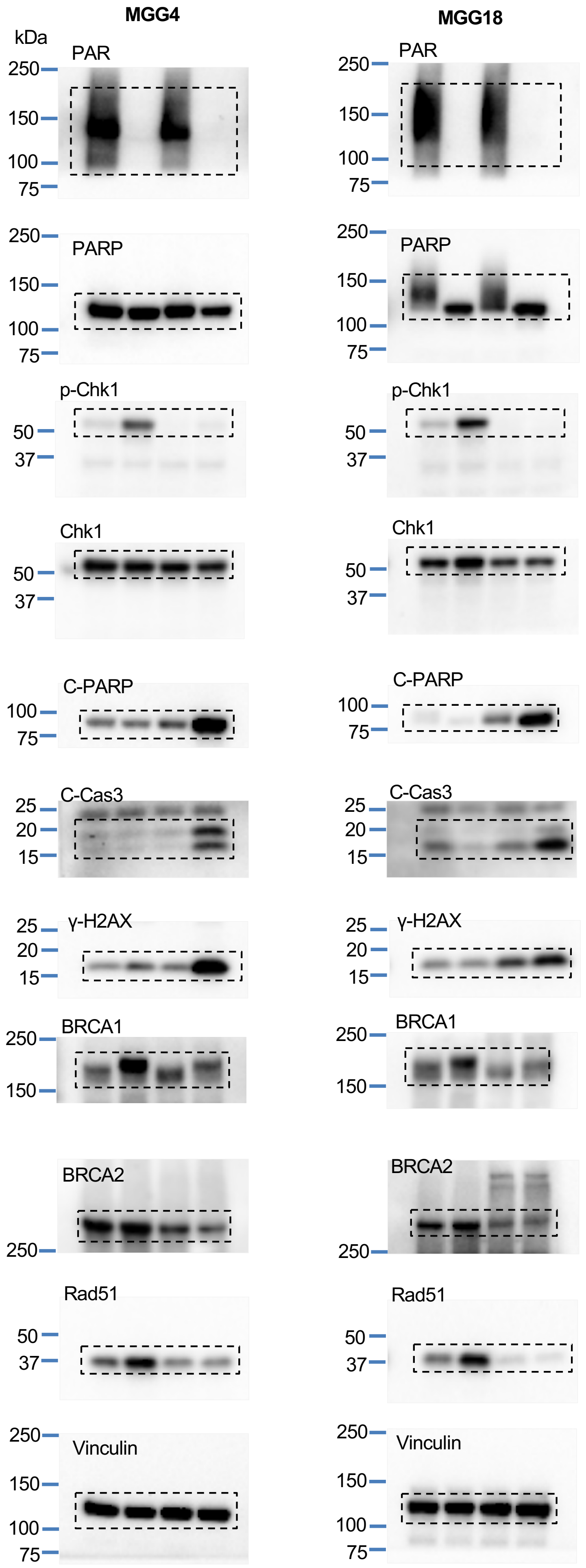

Source data 8. Uncropped western blots for Fig7e. Dotted lines indicate cropped area.

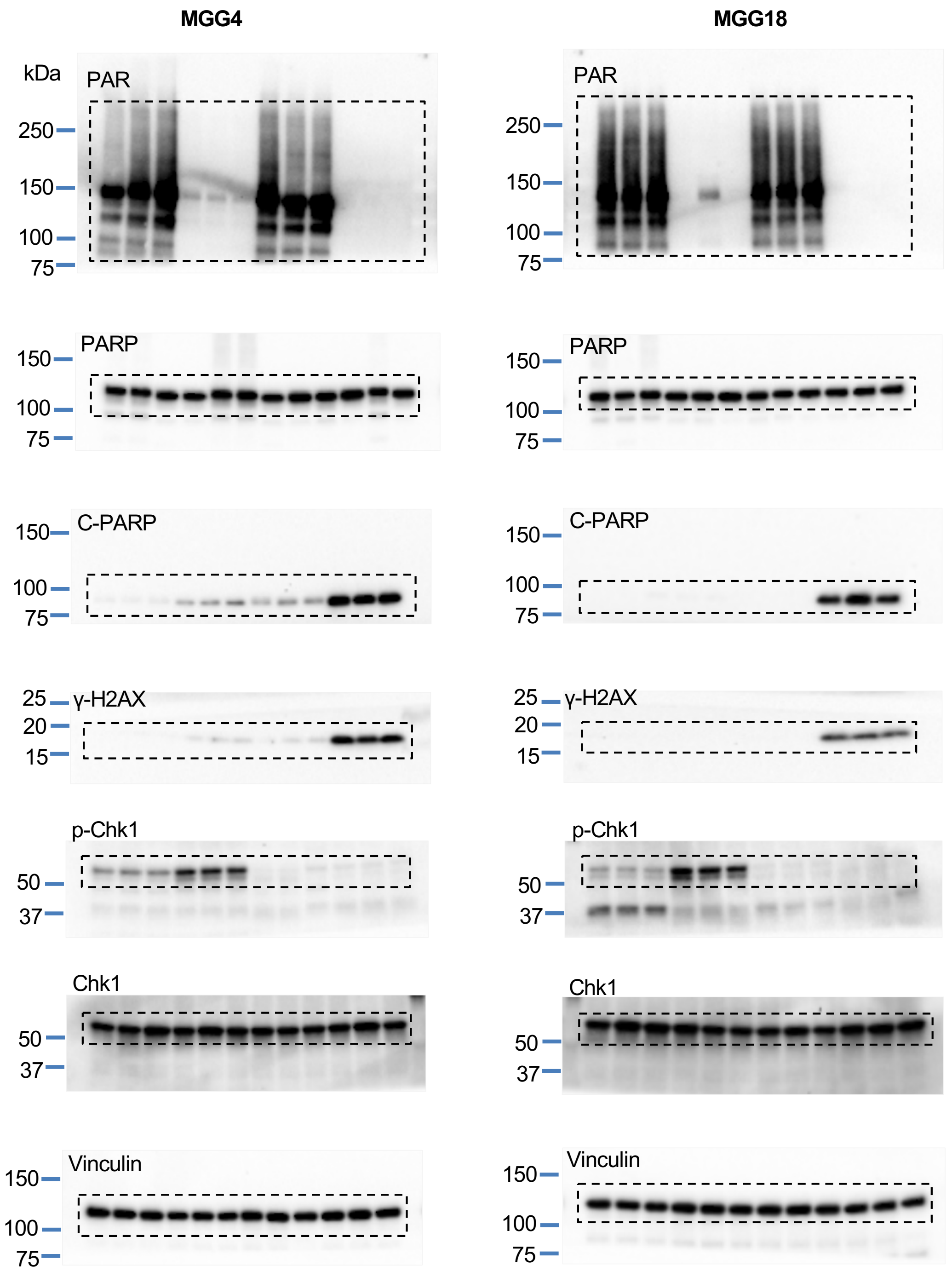

Source data 9. Uncropped western blots for Fig8e. Dotted lines indicate cropped area.

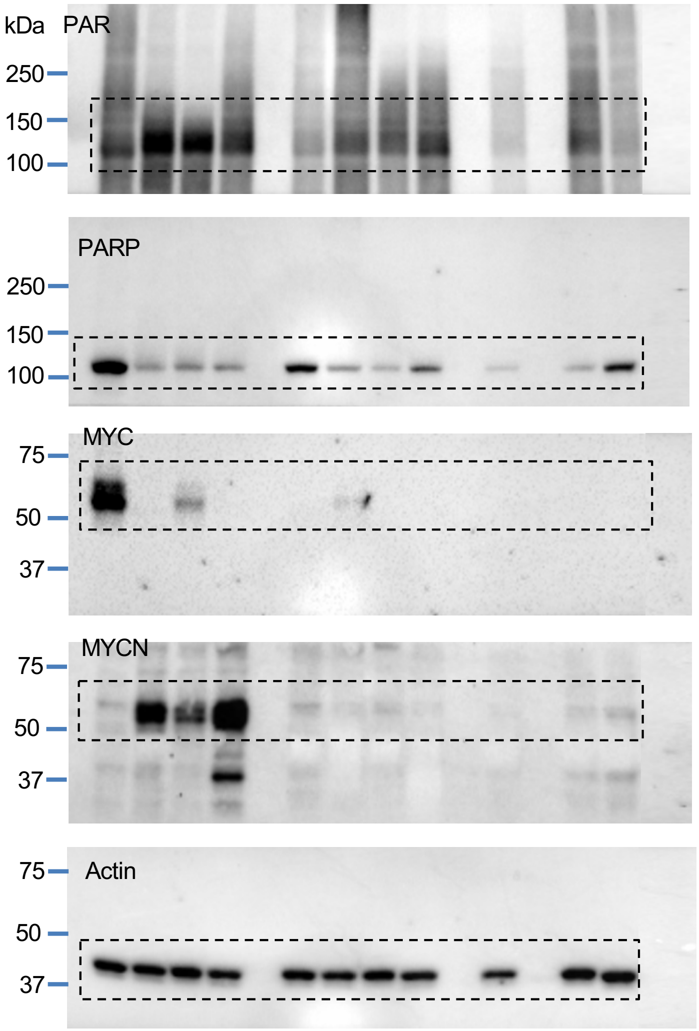

Source data 10. Uncropped western blots for Supplementary Fig 1a. Dotted lines indicate cropped area. Unrelated blots on the right are covered. Actin is the same blots as in **Source data 2**.
